# Molecular insights into DNA recognition and methylation by non-canonical type I restriction-modification systems

**DOI:** 10.1038/s41467-022-34085-z

**Published:** 2022-10-27

**Authors:** Jingpeng Zhu, Yina Gao, Yong Wang, Qi Zhan, Han Feng, Xiu Luo, Peipei Li, Songqing Liu, Hai Hou, Pu Gao

**Affiliations:** 1 CAS Key Laboratory of Infection and Immunity, CAS Center for Excellence in Biomacromolecules, Institute of Biophysics, Chinese Academy of Sciences, Beijing, 100101 China; 2 National Laboratory of Biomacromolecules, CAS Center for Excellence in Biomacromolecules, Institute of Biophysics, Chinese Academy of Sciences, Beijing, 100101 China; 3 University of Chinese Academy of Sciences, Beijing, 100049 China; 4 Institute of Medical Research, Northwestern Polytechnical University, Xi’an, 710072 China; 5 Science and Technology Innovation Center, Shandong First Medical University & Shandong Academy of Medical Sciences, Jinan, 250000 China

**Keywords:** Enzyme mechanisms, Structural biology

## Abstract

Type I restriction-modification systems help establish the prokaryotic DNA methylation landscape and provide protection against invasive DNA. In addition to classical m6A modifications, non-canonical type I enzymes catalyze both m6A and m4C using alternative DNA-modification subunits M1 and M2. Here, we report the crystal structures of the non-canonical PacII_M1M2S methyltransferase bound to target DNA and reaction product S-adenosylhomocysteine in a closed clamp-like conformation. Target DNA binds tightly within the central tunnel of the M1M2S complex and forms extensive contacts with all three protein subunits. Unexpectedly, while the target cytosine properly inserts into M2’s pocket, the target adenine (either unmethylated or methylated) is anchored outside M1’s pocket. A unique asymmetric catalysis is established where PacII_M1M2S has precisely coordinated the relative conformations of different subunits and evolved specific amino acids within M2/M1. This work provides insights into mechanisms of m6A/m4C catalysis and guidance for designing tools based on type I restriction-modification enzymes.

## Abstract (web summary)

Type I R-M systems help establish the prokaryotic DNA methylation landscape and provide protection against invasive DNA. Here, the authors report on detailed structural and molecular mechanisms of a non-canonical type I R-M methyltransferase.

## Introduction

Restriction-modification (R-M) systems are found in over 95% of bacterial and archaeal genomes, where they provide a major defense against phages and invasive DNA^1–3^. R-M systems are comprised of two contrasting enzymatic activities: a methyltransferase (MTase) and a restriction endonuclease (REase). The REase cleaves foreign DNA at specific sites, while MTase activity ensures discrimination between self and nonself DNA by adding methyl groups to the same specific DNA sequence within the host genome^1,4^. In addition to antiphage defense, MTases within R-M systems also play other roles such as establishing the DNA methylation landscape of prokaryotes^5^, repairing DNA mismatch^6^, and regulating gene expression^7^. R-M systems are classified mainly into four different types (I–IV) based on their subunit composition, cofactor requirements, and DNA cleavage properties^4,8^. Type I R-M systems are multi-subunit molecular machines composed of separate DNA-specificity subunit (S), DNA-modification subunit (M), and DNA-restriction subunit (R)^4,9,10^. These three subunits can assemble into two types of complexes: the M_2_S MTase complex with solely methyltransferase activity, and the R_2_M_2_S REase complex with both methyltransferase and endonuclease activities^4^. Characteristically, type I MTases recognize bipartite DNA motifs comprising two specific sequences separated by a non-specific spacer (e.g., the EcoKI recognition sequence is 5'-AAC-NNNNNN-GTGC-3'), and methylate a base in each specific motif, resulting in one methylated base on each strand^11,12^.

It has long been known that the type I R-M systems utilize two identical copies of a single M subunit to form the M_2_S MTase complex, which exclusively generates m6A modifications on both DNA strands^4,12^. A paradigm shift away from this principle came with the discovery of a non-canonical subgroup of type I R-M enzymes from many bacterial species that possess two different M subunits and catalyze m6A modification on one strand and m4C modification on the other^12^. The different M subunits group into two families, with one (termed as M1) having an ‘NPPF’ catalytic motif and generating m6A modifications, and the other (termed as M2) having an ‘NPPY’ catalytic motif and generating m4C modifications^12^. The protein components within this non-canonical subgroup of type I systems share high degree of similarities, suggesting that they may have recently evolved from the more common classical m6A type I systems^12^. While the discovery of novel m6A/m4C type enzymes broadens our understanding of DNA recognition and modification by type I R-M systems, the detailed molecular and structural mechanisms of complex assembly, DNA recognition, and DNA modification are still unclear.

In addition to the lack of any structural information of novel m6A/m4C systems, high-resolution structures of classical type I systems have also been difficult to obtain^13,14^, despite being studied for over five decades^15,16^. It is only recently that the crystal structures of either individual subunits^17–23^ or the M_2_S MTase complex in the absence of target DNA^24^ have emerged. Our recent cryo-electron microscopy work^25^ of the classical type I EcoR124I system reports the conformational dynamics of these micromolecular machines, but does not provide atomic or near-atomic densities for the core M_2_S MTase complex in its target DNA-bound conformation. To better understand the assembly and mechanism of non-canonical type I MTases, we determined the crystal structures of the representative PacII MTase complex bound to target DNA (either unmethylated or carrying m6A methylation) and reaction product S-adenosylhomocysteine (SAH). These structures, together with extensive mutational analysis, reveal a number of unexpected features of the non-canonical type I MTase and provide detailed explanations for many previous functional observations^12^. Furthermore, this work also provides guidance for the future design of molecular biology tools based on type I MTases.

## Results

### Overall structure of PacII_M1M2S MTase bound to DNA and SAH

**Fig. 1:**
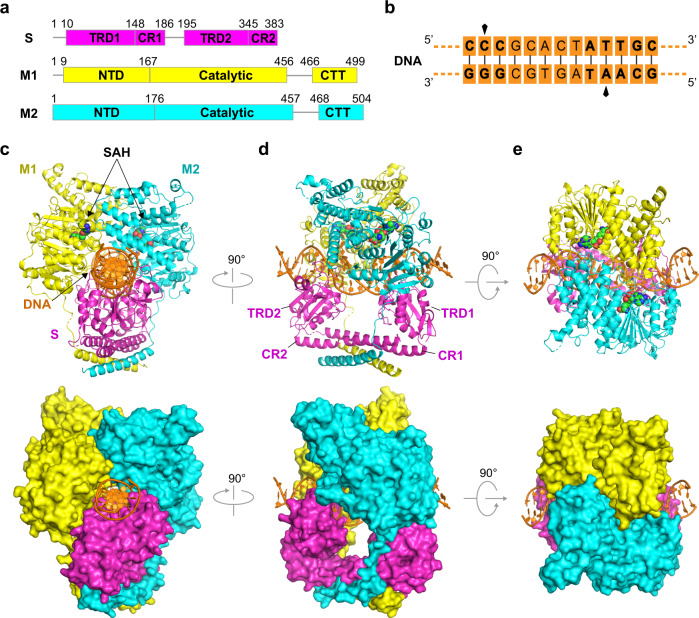
Overall structure of PacII_M1M2S heterotrimer complex bound to target DNA and SAH. **a** Domain organization of S, M1, and M2 subunits of PacII MTase, with domain boundary labeled according to this work. **b** Schematic representation of the target DNA used for co-crystallization. The bipartite recognition motifs (5'-CCC-3' and 5'-GCAAT-3') are shown in bold with the cytosine and adenine for methylation indicated by black arrows. **c**–**e** The side view (**c**), front view (**d**), and top view (**e**) of M1M2S-DNA-SAH complex in ribbon (up) and surface (down) representations. Proteins and DNA are color-coded as in (**a**) and (**b**), SAH molecules are shown in green spheres.

We purified the full-length PacII_M1M2S MTase (Fig. 1a) using a co-expression strategy in *E. coli* (Supplementary Fig. 1a) and confirmed that this sample can actively add m6A and m4C modifications onto its target DNA sequence based on mass spectrometry and kinetic assays (Fig. 1b; Supplementary Fig. 1b–e). To understand the molecular mechanism of DNA methylation by this novel type I MTase, we determined the 2.4 Å crystal structure of PacII_M1M2S heterotrimer bound to a 25 bp unmethylated target DNA and the reaction product SAH (Fig. 1c–e; Supplementary Table 1). The asymmetric unit contains one M1M2S heterotrimer, one DNA duplex, and two SAH molecules (Fig. 1c; Supplementary Fig. 1f, g). There are a few disordered regions for which we did not build the model, including the amino acids 1-9/189-192 of the S subunit, 461-464/498-499 of the M1 subunit, and 61-64/501-504 of the M2 subunit. The overall structure adopts a compact and closed clamp-like conformation, with the DNA passing through a central tunnel formed by all three protein subunits (Fig. 1c–e). The two different M subunits have a similar domain composition containing an N-terminal domain (NTD), a Catalytic domain (CD), and a C-terminal tail (CTT). Although the individual domains of M1 and M2 share similar folds, the relative inter-domain conformations show considerable differences for the two subunits (Supplementary Fig. 2a–c). Similar to classical type I R-M systems, the S subunit of PacII is composed of two specific-recognition domains (TRD1 and TRD2) for recognizing the bipartite DNA sequences, and a coiled-coil conserved region (CR1 and CR2) that separates the TRDs (Fig. 1d; Supplementary Fig. 3a–c). TRD1 and TRD2 bind to 5'-CCC-3' and 5'-GCAAT-3' motifs of the target DNA, respectively (Supplementary Fig. 3a). Thus, each TRD positions the bound motifs to the adjacent M2 and M1 subunits, respectively (Fig. 1c–e). Notably, the target adenine and cytosine bases have flipped out from the DNA duplex (Supplementary Figs. 1f, 3a). Given that the previous closed-state structures of classical type I R-M systems only provide low-quality local electron densities for the MTase region^14,25^, the current high-resolution structure provides an opportunity to discuss detailed inter-molecule contacts of type I MTases (for both novel and classical systems) in the closed-state.

### Assembly of PacII_M1M2S heterotrimer in closed-state

**Fig. 2:**
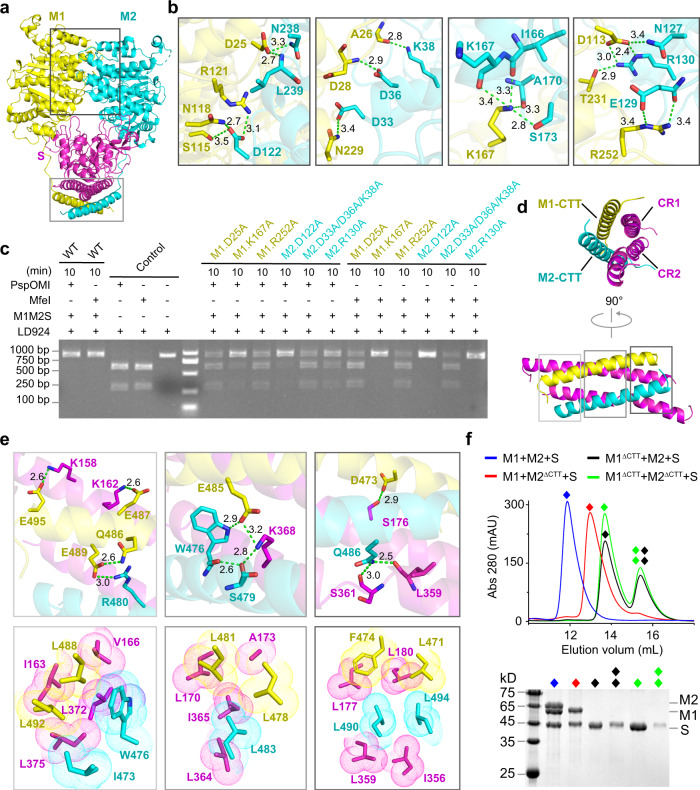
Intermolecular interactions of PacII_M1M2S heterotrimer complex. **a** Overall view of M1-M2 interface and four-helix bundle interface. **b** Detailed interactions of M1-M2 interface. Green dashed lines indicate hydrogen bonds. **c** PspOMI/MfeI-digestion analysis of mutations within M1-M2 interface. **d** The Four-helix bundle interface formed by CR1 and CR2 of S subunit, and CTTs of both M subunits as show in (**a**). **e** Hydrogen bonding (up) and hydrophobic (down) interactions within the four-helix bundle as show in (**d**). Green dashed lines indicate hydrogen bonds. **f** Assembly analysis of M1M2S heterotrimer complex with CTT deletion of M1 and/or M2. Elution profiles of size-exclusion chromatography and SDS-PAGE results for indicated samples. Note that the S subunit alone exhibits a dynamic equilibrium of monomer and dimer in size-exclusion chromatography. Experiments in **c**, **f** were repeated independently three times with similar results. Source data are provided as a Source Data file.

There are two major protein-protein interfaces in the current structure: an M1-M2 interface formed by the NTD-Catalytic dual domains of both M subunits, and a four-helix bundle interface formed by the coiled-coil conserved region of S subunit and C-terminal tails of both M subunits (Fig. 2a). The M1-M2 interface covers a 1325 Å^2^ area and is mainly mediated by hydrogen-bonding contacts (Fig. 2b). Distinct from the four-helix bundle interface which exists in both the previous open state^24^ and the current close state, this M-M interface is formed when target DNA is bound (Supplementary Fig. 3d, e). We generated different mutations at this interface and designed an easy-to-operate PspOMI/MfeI-digestion assay. A linear DNA (924 bp; termed as LD924) containing three PacII recognition sites was recognized and methylated by PacII_M1M2S, with unmethylated DNA subsequently being digested by PspOMI and MfeI endonucleases respectively (Supplementary Fig. 4a). Compared with wild-type PacII MTase that completely blocks PspOMI/MfeI-digestion, mutations of M1 (D25A, R252A) and M2 (D33A/D36A/K38A, R130A) at the M1-M2 interface show reduced methylation activities, albeit to different extents (Fig. 2c). Thus, the contacts between NTD-Catalytic dual domains of both M subunits are critical for the methylation of target DNA.

The four-helix bundle interface is composed of extensive hydrophobic and hydrogen-bonding interactions (Fig. 2d, e). Given that this interface has also been observed in the open state of the classical type I MTase^24^, the four-helix bundle may represent a critical anchor region in both open and closed conformations of type I R-M systems. Indeed, deletion of the CTTs of M1 and M2 completely disrupts the assembly of M1M2S heterotrimer (Fig. 2f). Previous sequencing results^12^ have shown that expression of just M1 with S retains partial m6A modification activity, while expression of M2 alone with S results in no detectable modification. We anticipated that M1 alone, but not M2 alone, may still be able to bind S. To test this hypothesis, we co-expressed M1 with S or M2 with S and purified the samples by using an identical strategy as for the wild-type PacII MTase. Interestingly, M1 still binds S but only forms a partial MTase containing one M1 and one S, while M2 could not interact with S to form a complex (Supplementary Fig. 4b). Furthermore, CTT deletion of M1 abolishes the assembly of the whole complex, while CTT deletion of M2 does not affect the formation of M1S heterodimer (Fig. 2f). These results indicate that the binding between M1 and S is the first step of complex assembly, which provides a platform to recruit M2 and form the fully functional PacII_M1M2S MTase.

In addition to the above two major interfaces, TRD2 and TRD1 of S also form several hydrogen bonds with the adjacent M1 and M2 subunits, respectively (Supplementary Fig. 4c, d). Interestingly, we also observed some interactions between M2 and TRD2 in the closed conformation, but not for M1 and TRD1 (Supplementary Fig. 4c, d). Therefore, we generated two mutants of S to disrupt the M1-TRD2 (E318A/N327A) and M2-TRDs (N121A/E128A/K136A/R214A) interfaces, and found that the M2-TRDs interface is more important for DNA binding and methylation activity (Supplementary Fig. 4e, f).

### DNA recognition by S subunit

**Fig. 3:**
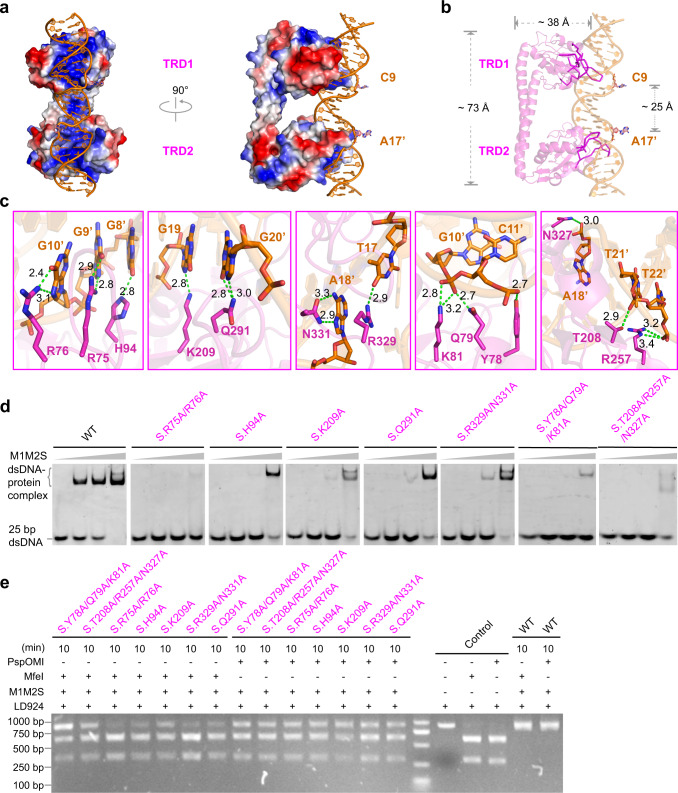
Specific recognition of target DNA by S subunit of PacII MTase. **a** Electrostatic surface of S subunit bound to target DNA, where the red indicated for negative and blue for positive charges. **b** Cartoon representation of S subunit bound to target DNA, with outward-loops of TRD1/TRD2 insert into the grooves of bipartite DNA. **c** Detailed specific and non-specific interactions between the loop regions of TRD1/TRD2 and target DNA. Green dashed lines indicate hydrogen bonds. **d** Electrophoretic mobility shift analysis of related mutations as show in (**c**). **e** PspOMI/MfeI-digestion analysis of mutations within S-DNA interface as show in (**c**). Experiments in **d**, **e** were repeated independently three times with similar results. Source data are provided as a Source Data file.

As the DNA recognition subunit, S forms a large interface (2071 Å^2^) with the target DNA (Fig. 3a, b). TRD1 and TRD2 form extensive hydrogen-bonding interactions with both backbone and bases of the target DNA (Supplementary Fig. 5). The two recognition motifs (5'-CCC-3' and 5'-RCAAY-3') of DNA bind tightly within the positively charged grooves of TRD1 and TRD2 (Fig. 3a). The coiled-coil region of S forms no contact with DNA and plays the role of a ruler (~73 Å), to determine that the distance between TRD1 and TRD2 matches the spacer length of target DNA bases (~25 Å) (Fig. 3b). The DNA-interacting amino acids are mainly distributed in the loop regions, which not only provide the conformational flexibility for protein-DNA interactions, but also allows TRDs to tolerate different evolutionary mutations without affecting the protein folding (Fig. 3b and Supplementary Fig. 3a–c). This structural feature ensures the DNA recognition diversity of type I R-M systems, and provides a basis for designing new TRDs with specific DNA recognition properties. To establish the specificity of DNA recognition, several critical amino acids within TRD1 (R75, R76, H94) and TRD2 (K209, A272, Q291, R329, N331) form direct contacts with the bases (or paired bases) of 5'-CCC-3' and 5'-GCAAT-3' motifs. TRD1 (Y78, Q79, K81) and TRD2 (T208, R257, N327) also form non-specific interactions with the DNA backbone (Fig. 3c). We generated multiple mutations and performed both electrophoretic mobility shift assay (EMSA) and PspOMI/MfeI-digestion experiments to validate these interactions. As expected, these mutations notably reduce both DNA binding and DNA methylation abilities of PacII MTase (Fig. 3d, e), highlighting the critical roles of interactions between S and DNA.

### Interactions between DNA and M1/M2 subunits

**Fig. 4:**
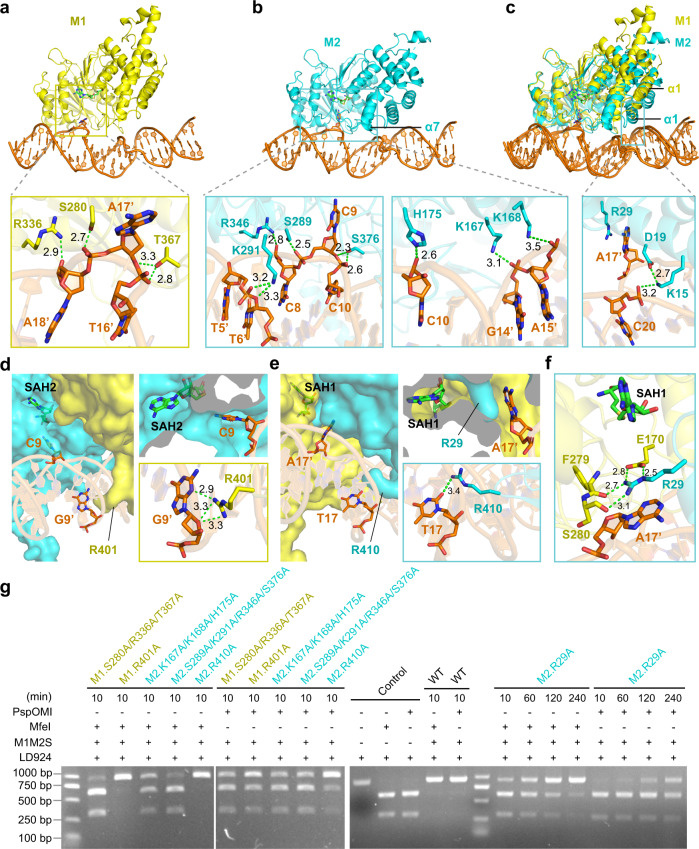
Detailed interactions between DNA and M1/M2 subunits of PacII MTase. **a, b** Overall structures and detailed interactions of M1-DNA interface (**a**) and M2-DNA interface (**b**). Green dashed lines indicate hydrogen bonds. **c** Structural superimposition of M1-DNA and M2-DNA by using DNA as reference, and the helix α1 of M2 insert into the major groove of DNA to mediate contacts. Green dashed lines indicate hydrogen bonds. **d** Surface view of M1/M2 bound to DNA motif (5'-CCC-3'), target cytosine (C9) inserts into the catalytic pocket of M2, and residue R401 of M1 contacts with the paired guanine (G9'). Green dashed lines indicate hydrogen bonds. **e** Surface view of M1/M2 bound to DNA motif (5'- GCAAT −3'), target adenine (A17') is blocked by residue R29 of M2, and the paired thymine (T17) contacts with residue R410 of M2. Green dashed lines indicate hydrogen bonds. **f** Residue R29 of M2 formed hydrogen bonds (green dashed lines) with residues E170, F279, and S280 of M1, and blocked target adenine (A17') from entering the catalytic pocket of M1. **g** PspOMI/MfeI-digestion analysis of mutations within M1-DNA and M2-DNA interfaces. The experiment was repeated independently three times with similar results. Source data are provided as a Source Data file.

In addition to S, M1 and M2 also form extensive hydrogen-bonding and stacking interactions with both backbone and specific bases of the target DNA (Supplementary Fig. 5). Surprisingly, the interface between M2 and DNA (1152 Å^2^) is significantly larger than that of M1 and DNA (680 Å^2^) (Fig. 4a, b). Structural superimposition between M1-DNA and M2-DNA, using DNA as reference, shows that M2 adopts a more lying-down conformation toward DNA than that of M1 (Fig. 4c). This conformational difference allows M2, but not M1, to form more contacts with DNA through its α7-helix (K167 and K168) and α1-helix (K15, D19, and R29) (Fig. 4b, c). In line with these observations, this lying-down conformation of M2 also contributes to the interactions between its α1-helix (R11) and the distal TRD2 domain of S subunit (Supplementary Fig. 4d). The base-specific interactions between M1/M2 and DNA are mainly mediated by the flipped-out target adenine (A17') and cytosine (C9), as well as their originally paired thymine (T17) and guanine (G9') (Supplementary Fig. 5). Two arginine residues (R401 of M1 and R410 of M2) insert into the minor grooves of the distal DNA motifs and respectively interact with guanine (G9') and thymine (T17) (Fig. 4d, e), thus helping to stabilize the structure of the DNA duplex. To validate the importance of M1-DNA and M2-DNA interfaces, we generated multiple mutations to disrupt both non-specific backbone interactions (M1:S280A/R336A/T367A; M2:K167A/K168A/H175A and S289A/K291A/R346A/S376A) and base-specific interactions (M1:R401A; M2:R410A) (Fig. 4a–f). The PspOMI/MfeI-digestion assays show that these mutants notably reduce the methylation activity of PacII MTase (Fig. 4g).

Interestingly, although both target cytosine (C9) and adenine (A17') have flipped out from the DNA duplex, only cytosine C9 inserts into the catalytic pocket of the adjacent M2 subunit (Fig. 4d). The target adenine A17', however, is unexpectedly blocked by an arginine residue (R29) from M2, which forms a strong stacking interaction with adenine A17' and keeps it out of the M1 catalytic pocket (Fig. 4e). R29 is anchored at the entrance of M1 catalytic pocket by forming a stable hydrogen-bond network with E170, F279, and S280 from M1 (Fig. 4f). Moreover, sequence alignments show that this arginine residue is highly conserved in m4C MTase subunits (M2-type), but not m6A MTase subunits (M1-type), of the novel type I systems (Supplementary Fig. 6a; Supplementary Table 2). Notably, alanine substitution of R29 from M2 leads to reduced, rather than increased, m6A modification (Fig. 4g). Moreover, the R29A mutation also causes a strong reduction in m4C modification (Fig. 4g), although R29 does not form substantial contact with the 5'-CCC-3' motif. To better understand the functional relevance of R29-mediated contacts, we generated additional mutations, including E170A for M1 and A17'C for substrate DNA (the complementary T17 remains intact). PspOMI/MfeI-digestion results show that M1_E170A causes strong reductions for both m6A and m4C modifications (Supplementary Fig. 6b). In addition, mass spectrometry results show that the mutant DNA practically loses the modification at its A17'C site and also causes significant reduction for modification at the distal C9 site (Supplementary Fig. 6c). Taken together, the consistent methylation results for mutations of R29A (M2), E170A (M1), and A17'C (DNA) indicate that R29-mediated contacts may contribute to the coordination of current asymmetric conformation and the communication between subunits, rather than simply block the target adenine from entering M1’s pocket.

### Different conformations of M1 and M2 catalytic pockets

**Fig. 5:**
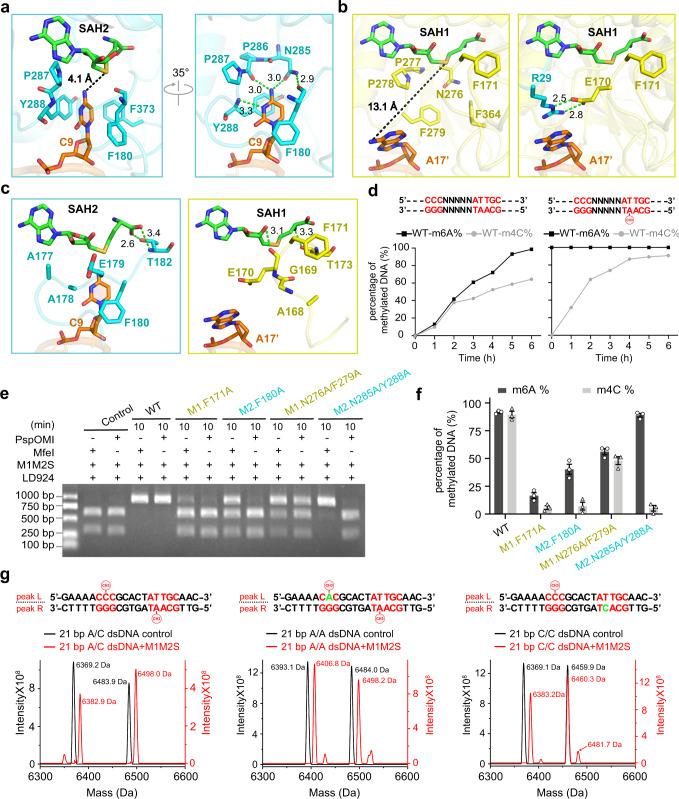
Catalytic pockets and structurally asymmetric methylation of PacII MTase. **a** Detailed interactions within the catalytic pocket of M2 subunit, the target cytosine (C9) points to the SAH2 (4.1 Å, black dashed line), form hydrogen bonds (green dashed lines) and stacking interactions with the catalytic motifs (NPPY/AAEF). **b** Detailed interactions within the catalytic pocket of M1 subunit, the target adenine (A17') is far from SAH1 (13.1 Å, black dashed line), and is blocked by residue R29 of M2 subunit. **c** The loop regions (M1: aa 164–174; M2: aa 173–183) adopt distinct orientations and form hydrogen bonds (green dashed lines) with SAH molecules. **d** Methylation activity analysis of unmethylated (left) and m6A hemi-methylated DNA (right) by wild-type enzyme at different time points (0–6 h), proportions of m6A and m4C methylated DNA were respectively counted and plotted. **e** PspOMI/MfeI-digestion analysis of mutations within the catalytic pockets of M1 (F171A, N276A/F279A) and M2 (F180A, N285A/Y288A) subunits at 10 min. **f** Proportions of methylated m6A and m4C DNA at 10 min were statistically counted to generate the histogram (*n* = 3). The mean ± SEM represents three independent determinations. Source data are provided as a Source Data file. **g** Mass spectrometry analysis of the DNA substrates with (red) or without (black) wild-type enzyme, the target bases of DNA substrates are A/C (left), A/A (middle), C/C (right) respectively.

The target cytosine (C9) is tightly buried within the M2 catalytic pocket and forms extensive stacking, hydrophobic, and hydrogen-bonding interactions with the protein (Figs. 4d, 5a). The residues F180, P287, Y288, and F373 adopt a compact aromatic cage to accommodate cytosine C9 (Fig. 5a). The nitrogen atom (N4) of cytosine C9 that needs to be methylated forms two hydrogen bonds with the side-chain of N285 and the main-chain of P286, thus ensuring that this N4 atom is in the proper position (Fig. 5a). Additional hydrogen bonds between N3 atoms of cytosine C9 and the main-chain of Y288 further stabilize the conformation of target cytosine (Fig. 5a). Notably, the N4 atom is in close proximity to the S-adenosylhomocysteine (SAH) molecule and points to the modeled donor-methyl group of S-adenosylmethionine (SAM) (Fig. 5a; Supplementary Fig. 6d). Given that the distance between N4 atom of cytosine to sulfur atom of SAH (4.1 Å) is slightly larger than the combination of the bond distances of donor-methyl (S-CH3 : ~1.8 Å) and acceptor-methyl (CH3-N : ~1.5 Å), further adjustments of the target cytosine and the enzyme pocket may be needed to achieve the methylation-competent conformation. Interestingly, since the target adenine (A17') is blocked by R29 of M2 and does not insert into M1 catalytic pocket (the distance between N6 atom of adenine and sulfur atom of SAH is 13.1 Å) (Fig. 4e; Fig. 5b), the compact aromatic cage found in M2 is not properly formed in M1 (Fig. 5a, b).

A loop region surrounding the catalytic pockets (M1: aa 164–174; M2: aa 173–183) adopts different conformations in M1 and M2, which in turn results in distinct orientations of the pocket-forming phenylalanine (F171 in M1; F180 in M2) (Fig. 5c). The conformational differences of this loop region also contribute to different hydrogen-bonding contacts between the protein and the SAH molecule (Fig. 5c). Taken together, these observations indicate that the catalytic pockets of M1 and M2 employ an induced-fit mechanism to recognize target bases and adopt different conformations with or without the bound bases.

### Structurally asymmetric methylation of m6A and m4C

The different interacting environments of the target adenine and cytosine suggest that the non-canonical m6A/m4C type I R-M systems may adopt a structurally asymmetric catalytic mechanism for m6A and m4C modifications (Fig. 5a–c). Given that the physiological substrates for type I MTases are hemi-methylated DNAs, we further determined the 2.6 Å crystal structure of PacII_M1M2S bound to SAH and a hemi-methylated target DNA carrying m6A modification (Supplementary Table 1). The overall structure of m6A hemi-methylated DNA-bound complex adopts a similar closed and asymmetric conformation to the unmethylated DNA-bound complex (Supplementary Fig. 7a). Moreover, while the target unmethylated cytosine is bound within M2’s pocket, the methylated adenine is still located outside M1’s pocket by forming the similar stacking interactions with R29 of M2 (Supplementary Fig. 7b–d). Together, these two structures indicate that the current asymmetric conformation likely represents a physiological relevant state of PacII MTase, rather than an artificial state due to the usage of unmethylated target DNA.

To explore whether the two M subunits can simultaneously adopt the lying-down or “productive” conformations, we modeled a lying-down structure for M1 based on the current structure of M2. The predicted model clearly shows that M1 and M2 cannot simultaneously stay in the lying-down conformation due to severe steric hindrance effects (Supplementary Fig. 7e). Thus, PacII_M1M2S will likely generate m6A and m4C modifications through separate and asymmetric steps, rather than simultaneously. Clearly, future structural determination of PacII_M1M2S bound to the hemi-methylated target DNA carrying m4C modification will be needed to further confirm the asymmetric methylation mechanism.

### Hemi-methylated DNA is a preferable substrate for PacII MTase

Type I R-M systems protect hemi-methylated host DNA through methylation and in the meantime remove unmethylated invading DNA through restriction, a process that is typically achieved by a preference of the MTase for hemi-methylated over unmethylated DNA substrates. To check whether PacII MTase also follows this principle, we measured the methylation rates of cytosine methylation by using either unmethylated or m6A hemi-methylated DNA substrate. As expected, the cytosine methylation rate for m6A hemi-methylated DNA is notably faster than that for non-methylated DNA (Fig. 5d). As a negative control, the pocket mutant of M2 (F180A) cannot catalyze m4C modification for both unmethylated and hemi-methylated substrates (Supplementary Fig. 8a, b). In addition, although the pocket mutant of M1 (F171A) can readily generate m4C modification for m6A hemi-methylated substrate (Supplementary Fig. 8b), m4C methylation for unmethylated substrate can only be detected when using a higher enzyme concentration (Supplementary Fig. 8a). Together, these results confirm that the m6A hemi-methylated DNA is a preferable substrate for the non-canonical PacII MTase. Based on the cytosine methylation results and the general substrate preference of type I MTases for hemi-methylated DNAs, it is reasonable to expect that PacII-mediated adenine methylation for m4C hemi-methylated DNA might also be more efficient than that for non-methylated DNA.

Interestingly, the PspOMI/MfeI-digestion and mass spectrometry assays both show that, when using the physiologically irrelevant unmethylated DNA as substrate, pocket mutations of M1 (F171A or N276A/F279A) affect the catalysis of not only m6A, but also m4C on the distal motif (Fig. 5e, f; Supplementary Fig. 8a, c). Conversely, pocket mutations of M2 (F180A or N285A/Y288A) significantly decrease the catalytic activity for m4C modification, while largely maintain the m6A generation (Fig. 5e, f; Supplementary Fig. 8a, c). It should be noted that these results do not suggest any strict sequential catalysis mechanism, given that the physiological substrates for PacII MTase are actually hemi-methylated host DNAs. Nevertheless, these assays may provide useful information for future tools development and in vitro enzymatic usage of PacII MTase.

### Selectivity of M1 and M2 catalytic pockets

Sequence alignments of different m6A/m4C type I R-M systems show that M1 subunits possess ‘AGEF’ and ‘NPPF’ motifs while M2 subunits possess ‘AAEF’ and ‘NPPY’ motifs within their catalytic pockets (Supplementary Fig. 9a and Table 2). However, it is unclear whether the different catalytic pockets contribute to the selectivity of m6A and m4C modifications. To better understand this question, we incubated the PacII MTase with an altered DNA substrate that carries a switching of target adenine and cytosine (Fig. 5g). Intriguingly, the mass spectrometry results show that the M2 subunit is able to catalyze both m4C and m6A modifications, while the M1 subunit can only modify m6A (Fig. 5g). Thus, the PacII_M1 subunit prefers to modify adenine, and PacII_M2 subunit has no rigorous selectivity for the m6A or m4C modification. These results are in line with the previous speculation that M2-type subunits may have recently evolved from the more common classical M1-type subunits^12^, that is, M2 still retains the original m6A modification activity after acquiring the new m4C modification ability.

### Proteins Ocr and ArdA inhibit PacII activity

It is known that phages and other mobile genetic elements have evolved different inhibitor proteins, represented by Ocr and ArdA, to overcome and inhibit the classical type I R-M systems^4,26,27^. To test whether Ocr and ArdA also bind to and inhibit the novel m6A/m4C type I systems, we purified both proteins and performed size-exclusion chromatography (SEC), electrophoretic mobility shift assay (EMSA), as well as PspOMI/MfeI-digestion assays (Supplementary Fig. 9b–d). Both Ocr and ArdA form stable complexes with PacII_M1M2S heterotrimer and compete for the binding of PacII_M1M2S to DNA, highlighting a DNA-mimicry strategy utilized by Ocr and ArdA (Supplementary Fig. 9b, c). Furthermore, PspOMI/MfeI-digestion assays confirm that Ocr and ArdA can efficiently inhibit the m4C and m6A methylation activities of PacII MTase (Supplementary Fig. 9d). Therefore, Ocr and ArdA could inhibit not only the classical type I R-M systems, but also the novel m6A/m4C systems.

## Discussion

Type I R-M systems were the first R-M systems to be discovered and have helped launch the molecular biology revolution. The identification of non-canonical m6A/m4C type I enzymes further broadens the scope of DNA modification and potential application of type I R-M systems. However, despite extensive attempts over five decades, no high-resolution structural information of the type I MTases bound to target DNA has been reported. Thus, a more detailed research of PacII MTase, shown in the current work, will be essential to understand the assembly and operation mechanisms of the non-canonical enzymes, as well as canonical type I R-M enzymes in general.

The current closed-state and previously reported open-state^24^ structures together provide an opportunity to discuss the conformational changes associated to the open-to-closed transition of type I MTases. Structural superimposition using the S subunits as references shows that, upon target DNA recognition, the NTD-Catalytic dual domains of M subunits will undergo significant rotation and movement toward the bound target DNA (Supplementary Fig. 3d, e). Given that non-canonical systems encode a typical DNA-restriction subunit (R) and are capable of regular phage restriction^12^, we anticipated that the stoichiometry of a fully assembled non-canonical holoenzyme will be R_2_M1M2S, similar to the R_2_M_2_S assembly of classical systems. Based on the current M1M2S structure and previously reported cryo-EM structures of the classical EcoR124I holoenzyme^25^, we were able to build potential models for the PacII holoenzyme in different conformational states (Supplementary Fig. 10a, b). Clearly, future structural determination of non-canonical holoenzymes in multiple conformations will be needed to better elucidate the assembly and operation mechanisms.

An unexpected finding of the current work is that PacII MTase utilizes a unique asymmetric methylation mechanism to generate m6A and m4C modifications. In our current two structures, the target cytosine within 5'-CCC-3' motif properly inserts into the catalytic pocket of M2, while the target adenine (unmethylated or m6A-methylated) within 5'-GCAAT-3' motif is blocked at the entrance of M1’s catalytic pocket. Since M1 and M2 will generate severe steric hindrance effects when simultaneously stay in the lying-down conformation, PacII_M1M2S will likely generate m6A and m4C modifications through separate and asymmetric steps, rather than simultaneously. Since that hemi-methylated DNAs are the physiological and preferable substrates for PacII MTase, such a unique asymmetric structural feature may provide a control of proper binding and methylation of the unmethylated target base on one strand by the methylation status of the other strand. Future structure determination of PacII MTase bound to m4C hemi-methylated DNA and related enzymatic analysis will further verify this hypothesis.

To maintain the current asymmetric methylation state, the PacII MTase complex needs to precisely coordinate the relative spatial positions of different subunits. Specifically, M2 needs to adopt a more lying-down conformation toward target DNA than that of M1, which ensures that M2 can form more contacts with both the adjacent 5'-CCC-3' motif and the distal 5'-GCAAT-3' motif. A special arginine residue of M2 (R29) forms stable stacking interactions with the target adenine and keeps it out of M1 catalytic pocket. R29-mediated interactions may also contribute to the coordination of current asymmetric conformation and the communication between subunits. This arginine residue is highly conserved within M2 subunits, but not M1 subunits, indicating that asymmetric catalysis might be a conserved mechanism for the novel m6A/m4C type I R-M systems. Interestingly, sequence alignments show that the arginine residue is also relatively conserved (about 80%) in the classical m6A/m6A type I MTases (Supplementary Fig. 11 and Table 3). Thus, the two identical M subunits of classical systems may also utilize a similar methylation mechanism for DNA recognition and m6A catalyzation. Further determination of high-resolution structures of classical MTases will be needed to answer this question.

In contrast to the enormously useful type II R-M enzymes, the type I systems have not been fully developed as common molecular biological tools. This situation has been on the verge of change due to the application of Single-Molecular-Real-Time (SMRT) DNA sequencing that enables direct identification of new type I R-M systems, as well as the recent structural biology breakthroughs that provide detailed mechanistic understanding of these sophisticated machines. Given that the S-DNA interactions are mainly mediated by several outward-protruding loops within two TRDs, it will be possible to design new enzymes with desired DNA specificities by only modifying these loop regions, rather than replacing the whole TRDs. Moreover, it is equally important to maintain other critical inter-domain interactions for enzyme engineering, such as the S-M four-helix bundle interface, the M-M interface, as well as the M-DNA interfaces. In addition, for the novel m6A/m4C type I enzymes, an intact asymmetric catalytic mechanism will also be important.

In summary, our work reveals detailed structural and molecular mechanisms of the non-canonical m6A/m4C modifications catalyzed by PacII_MTase. This work may also serve as an important reference in designing novel tools based on the type I R-M enzymes.

## Methods

### Construction of expression vectors

The genes encoding the HsdM1, HsdM2, and HsdS subunits of PacII_M1M2S (*Pseudomonas alcaligenes* NEB585) and those for the anti-restriction proteins Ocr and ArdA were codon-optimized for expression in *Escherichia coli* BL21 and synthesized at Invitrogen. The full-length M1 was cloned into the MCS1 (BamHI/NotI) of modified pRSFDuet1 (Invitrogen) with N-terminal 6His-SUMO tag that can be cleaved by ULP1 protease, and the full length of S was inserted into the MCS2 (NdeI/XhoI) of the same vector without a tag. The full-length M2 was cloned into the MCS1 (NcoI/XhoI) of pETDuet-1 (Invitrogen) without a tag. Additionally, Ocr and ArdA were cloned into the MCS1 (BamHI/NotI) of pRSFDuet1 bearing N-terminal 6His-SUMO tag, respectively. For the assembly analysis of PacII_M1M2S complex, we deleted the N-terminal 6His-SUMO tag of M1 and added 6His tag to the C-terminal of S subunit. Other point mutants and truncations of PacII_M1M2S were generated from the native construct by standard PCR methods, and sequences were subsequently confirmed by sequencing.

### Protein expression and purification

The PacII_M1M2S complex was co-expressed in *Escherichia coli* BL21 (DE3) cell strain, while Ocr or ArdA was overexpressed in *Escherichia coli* BL21 (DE3) alone. The cells were grown at 37 °C until OD_600_ reached 0.8 and the temperature was then cooled to 20 °C. Protein expression was induced by adding isopropyl-beta-D-thiogalactopyranoside (IPTG) at a final concentration of 0.2 mM and cells were harvested after 12 h. Cell pellets were resuspended in lysis buffer (50 mM Tris pH 7.5, 500 mM NaCl, 20 mM imidazole, 5% glycerol, 2.5 mM β-ME) and then disrupted by French Press. Lysates are centrifugated at 25,000 × g for 30 min and the supernatant was loaded onto a Ni-NTA column. The column was washed with lysis buffer and then eluted with the elution buffer (50 mM Tris pH 7.5, 300 mM NaCl, 250 mM imidazole, 5% glycerol).

Eluted PacII_M1M2S protein was treated with ULP1 protease for 8 h at 4 °C, diluted with buffer A (20 mM Tris pH 7.5, 100 mM NaCl) and loaded onto the HiTrap Heparin HP column (GE Healthcare). PacII_M1M2S complex was eluted with a linear gradient (0.1–1.0 M) of NaCl, followed by size-exclusion chromatography (SEC) on a Superdex G200 16/60 column (GE Healthcare) in buffer B (20 mM Tris pH 7.5, 300 mM NaCl, 1 mM DTT). The Se-methionine substituted PacII_M1M2S complex was purified in the same way. Lastly, PacII_M1M2S complex was concentrated to 20 mg/mL and stored in −80 °C. All mutants were purified following the same protocol used for preparation of wild-type enzyme. The samples for assembly analyses were purified like wild-type enzyme except for ULP1 cleavage.

Eluted Ocr and ArdA from Ni-NTA were digested by ULP1 protease for 8 h at 4 °C, diluted with buffer A (20 mM Tris pH 7.5, 100 mM NaCl) and loaded onto a HiTrap Q HP column (GE Healthcare). After a linear gradient elution and concentration of the target proteins, we proceeded to further purify on a Superdex G200 16/60 column (GE Healthcare) that was equilibrated with buffer C (20 mM Tris pH 7.5, 150 mM NaCl, 1 mM DTT). Lastly, Ocr or ArdA was concentrated to 30 mg/mL and stored in −80 °C. We evaluated the binding between PacII_M1M2S and Ocr (M1M2S:Ocr = 1:5, molar ratio) or ArdA (M1M2S:ArdA = 1:5, molar ratio) by size-exclusion chromatography (SEC) on a Superdex G200 16/60 column (GE Healthcare) in buffer C (20 mM Tris pH 7.5, 150 mM NaCl, 1 mM DTT).

### Crystallization and structure determination

For crystallization of PacII_M1M2S-DNA-SAH and PacII_M1M2S-DNA (m6A) -SAH complexes (10 mg/mL), we incubated the sample at a molar ratio of M1M2S:DNA (unmethylated or m6A methylated):SAH = 1:1.3:5 in buffer D (20 mM Tris pH 7.5, 150 mM NaCl) for 30 min and then proceeded for crystal screening. The crystals of PacII_M1M2S-DNA-SAH complex were generated and optimized by hanging drop vapor diffusion method at 20 °C, from drops containing solution buffer (0.1 M Imidazole pH 6.9, 40% PEG400). Se-methionine substituted crystals were grown under the same condition. The crystals of PacII_M1M2S-DNA (m6A) -SAH complex were generated and optimized from drops containing solution buffer (0.15 M di-ammonium hydrogen phosphate, 25% PEG3350) at 20 °C. All the diffraction data sets were collected at the Shanghai Synchrotron Radiation Facility (SSRF), and were indexed, integrated, and scaled by the HKL2000 program suite^28,29^ in BL18U. Single-wavelength Anomalous Dispersion (SAD) program in PHENIX^30^ was used to resolve the experimental phase of Se-PacII_M1M2S-DNA-SAH complex. Molecular Replacement (MR) program was used to resolve the experimental phase of PacII_M1M2S-DNA (m6A) -SAH complex. Model building and structural refinement of the structure were carried out by COOT^31^ and PHENIX, respectively. The statistics of the data collection and refinement are shown in Supplementary Table 1. Interfaces were analyzed in PISA 1.48^32^ and CCP4 Contact v8.0^33^. All structure figures were visualized with PyMOL (v.2.4).

### Kinetic assay of PacII MTase

The DNA methylation activity of PacII_M1M2S was measured by MTase-Glo^TM^ methyltransferase assay (Promega Cat#V7601)^34^. In this assay, the generated SAH (product of SAM) was converted to ATP, and then was detected via a luciferase reaction. We optimized the reaction conditions for pH, ionic strength, temperature, reaction time, enzyme concentration^35^, and determined the optimal reaction conditions (50 mM Tris pH 7.5, 100 mM NaCl, 26 °C, 5 min, [E] = 100 nM). To measure the *K*_m_ and *k*_cat_ for SAM (Supplementary Fig. 1c, e), we mixed 100 nM enzyme, 2 µM DNA and varying concentrations of SAM (0 to 190 µM) in a 20 µL reaction. Reactions were carried out in optimal conditions and terminated by heating at 75 °C for 10 min. Then, we added 8 µL of the reaction mixture to the 384-well plate for luciferase reaction and measured luminescence signal using a Varioskan Flash Multimode Reader (Thermo scientific). To measure the *K*_m_ and *K*_cat_ for DNA (Supplementary Fig. 1d, e), we carried out reactions with 100 nM enzyme, 50 µM SAM and varying concentrations of DNA (0 to 4 µM) in optimal conditions, post-treatments were as same as for SAM. Data were collected and both curves were fitted in GraphPad Prism 8.3.

### Mass spectrometry

The target DNA (target bases were A/C) used for methylation activity assay was mixed with wild-type enzyme (M1M2S:DNA:SAM = 1:10:100, molar ratio, [M1M2S] = 100 nM) in a 20 µL methylation buffer (20 mM Tris pH 7.5, 100 mM NaCl). The reaction was set up at 26 °C for 24 h and stopped by heating at 75 °C for 10 min, then the mixture was diluted to 200 µL with ddH_2_O and subjected to the Finnigan LCQ Advantage MAX (Thermo Fisher Scientific). We collected MS spectra data and fitted to the curves (Supplementary Fig. 1b). The swapped DNA substrates (target bases were A/A or C/C) used for methylation selectivity assays were treated as same as for the methylation activity assay (Fig. 5g). The original DNA (target bases were A/C) and swapped DNA (target bases were C/C, equal to A17'C mutation) were also used for functional relevance assay of M2_R29 mediated interaction. We prepared samples (M1M2S:DNA:SAM = 1:100:400, molar ratio, [M1M2S] = 100 nM) in a 200 µL reaction, 20 µL mixture was taken out at different time points (0 h, 1 h, 2 h, 3 h, 4 h, 5 h, 6 h, 7 h, 8 h), post-treatments were as same as described above. We counted proportions of m6A and m4C methylated DNA at different time and fitted to curves using Graph Prism 8.3 (Supplementary Fig. 6c).

The mass spectrometry assay was applied to check whether PacII MTase preferred the hemi-methylated DNA (5'-GTCGAAAACCCGCACTATTGCAACAG-3' and 5'-CCTGTTGCAm6ATAGTGCGGGTTTTCGA-3'). We mixed unmethylated (target bases were A/C) or m6A hemi-methylated DNA (target bases were m6A/C) with wild-type enzyme (M1M2S:DNA:SAM = 1:100:400, molar ratio, [M1M2S] = 100 nM) in a 180 µL reaction, 20 µL mixture was taken out at different time points (0 h, 1 h, 2 h, 3 h, 4 h, 5 h, 6 h), post-treatments and data process were as same as described above (Fig. 5d). Some mutations of PacII_M1M2S (M1: F171A; M2: F180A) were also used for this assay (Supplementary Fig. 8a, b).

### Electrophoretic mobility shift assay

We performed the electrophoretic mobility shift assay to monitor the interaction between DNA and proteins. Target DNA substrates labeled with 5'FAM (5'-FamTCGAAAACCCGCACTATTGCAACAG-3' and 5'-CTGTTGCAATAGTGCGGGTTTTCGA-3') were synthesized at Invitrogen. Here, the purified proteins at concentrations of 0, 0.05, 0.1, and 0.4 µM were incubated with the dsDNA (0.1 µM) in a 20 µL reaction buffer (20 mM Tris pH 7.5, 150 mM NaCl, 5 mM MgCl_2_) at 4 °C for 30 min. The samples were augmented with 5 µL of EMSA loading buffer (50% glycerol, 10 mM Tris pH 7.5), then 10 µL of each sample was loaded onto a 6% acrylamide gel (29:1 acrylamide:bisacrylamide) and run in 0.5xTris-borate-EDTA (TBE) buffer (45 mM Tris pH8.0, 45 mM boric acid, 1 mM EDTA) for 25 min at 120 V. The DNA and shifted bands can be visualized by the FAM label after exposure. Moreover, the effect of inhibitor proteins (Ocr and ArdA) was also tested by the electrophoretic mobility shift assay. First, the wild-type PacII_M1M2S (0.2 µM) was pre-mixed with LD924 dsDNA (10 nM), and a gradient of inhibitor proteins (Ocr or ArdA: 0, 0.2, 0.4, 0.8 µM) was added. Shifted bands were visualized using the Goldview model after exposure.

### Cleavage assay of DNA methylated by PacII MTase

PacII REase cuts DNA at variable positions and produces discrete bands by agarose gel electrophoresis. To clearly evaluate the methylation activity of mutated PacII_M1M2S, we designed dsDNA (LD924) containing two reversely palindromic motif that can be specifically recognized by PacII_M1M2S (5'-GCAACACTGTGGGCCCGCACAATTGT-3') and restriction enzymes (PspOMI for 5'-GGGCCC-3' and MfeI for 5'-CAATTG-3', NEB) (Supplementary Fig. 4a). PspOMI and MfeI cut the substrate (LD924 without m6A or m4C modification) into two small fragments, respectively, while the methylated DNA will be resistant to the digestion. Samples for cleavage assays were prepared similar to Mass spectrometric analysis. The dsDNA (LD924) was mixed with candidate mutants of PacII_M1M2S (M1M2S:DNA:SAM = 1:1:100, molar ratio, [M1M2S] = 0.1 µM) in a 20 µL methylation buffer (20 mM Tris pH 7.5, 100 mM NaCl), and the reaction was set up at 26 °C for 10 min and stopped by heating at 75 °C for 10 min. We equally divided the 20 µL sample, added PspOMI and MfeI, respectively, and set up digestion at 37 °C for 1 h. Products were then loaded onto the 1% (m/v) agarose gel and run in 1xTris-acetate-EDTA (TAE) buffer (4 mM Tris pH8.5, 20 mM acetic acid, 1 mM EDTA) for 15 min at 120 V. The DNA bands were visualized using the Goldview model after exposure.

For time dependent cleavage assays, dsDNA (LD924) was mixed with mutant PacII MTase (M1:E170A; M2:R29A) (M1M2S:DNA:SAM = 1:1:100, molar ratio, [M1M2S] = 0.1 µM) in 40 µL methylation buffer (20 mM Tris pH 7.5, 100 mM NaCl). The reaction was set up at 26 °C and took out 10 µL reaction product at different time points (10 min, 60 min, 120 min, 240 min), post-treatments were as same as described above. Other mutants (M1:F171A, N276A/F279A; M2:F180A, N285A/Y288A) were treated similarly to R29A mutation above, we took out 10 µL reaction product at different time points (10 min, 20 min, 40 min). The brightness values of DNA bands on the agarose gel were recorded, and proportions of methylated DNA (undigested DNA) were counted to generate histogram in Graph Prism 8.3.

### Sequence alignment and analysis

Amino acid sequences of all previously characterized members of Type I R-M systems, containing classical m6A/m6A and novel m6A/m4C MTase, were taken from the REBASE database^2^ (http://rebase.neb.com/rebase/). The catalog was used for searches with PacII_M1M2S as queries, and 10 classical m6A/m6A or 10 novel m6A/m4C type I MTases were unbiasedly extracted from the top2000 results (Supplementary Tables 2, 3). All sequences were subsequently aligned using the Clustal Omega 1.2.1^36^. After the refinement of poorly aligned regions, manual modulations were introduced based on the secondary structure prediction and PSI-BLAST pairwise comparison. Evolutionary tree and sequence alignments were generated by MEGA X^37^ and ESPRIPT3.0^38^, respectively.

### Reporting summary

Further information on research design is available in the Nature Research Reporting Summary linked to this article.

## Supplementary information

Supplementary Information

Reporting Summary

## Data Availability

The data that support this study are available from the corresponding author upon request. The PDB accession numbers for the coordinates and structure factors reported in this paper are 7VRU PacII_M1M2S-DNA-SAH and 7VS4 [10.2210/pdb7VS4/pdb] PacII_M1M2S-DNA(m6A)-SAH. Source data are provided with this paper.

## Source data

Source Data

## References

[1] Oliveira PH, Touchon M, Rocha EP (2014). The interplay of restriction-modification systems with mobile genetic elements and their prokaryotic hosts. Nucleic Acids Res.

[2] Roberts RJ, Vincze T, Posfai J, Macelis D (2015). REBASE-a database for DNA restriction and modification: enzymes, genes and genomes. Nucleic Acids Res.

[3] Dy RL, Richter C, Salmond GP, Fineran PC (2014). Remarkable mechanisms in microbes to resist phage infections. Annu Rev. Virol..

[4] Loenen WA, Dryden DT, Raleigh EA, Wilson GG (2014). Type I restriction enzymes and their relatives. Nucleic Acids Res.

[5] Blow MJ (2016). The epigenomic landscape of prokaryotes. PLoS Genet.

[6] Modrich P, Lahue R (1996). Mismatch repair in replication fidelity, genetic recombination, and cancer biology. Annu Rev. Biochem.

[7] Casadesus J, Low D (2006). Epigenetic gene regulation in the bacterial world. Microbiol Mol. Biol. Rev..

[8] Roberts RJ (2003). A nomenclature for restriction enzymes, DNA methyltransferases, homing endonucleases and their genes. Nucleic Acids Res.

[9] Murray NE (2000). Type I restriction systems: sophisticated molecular machines (a legacy of Bertani and Weigle). Microbiol Mol. Biol. Rev..

[10] Youell J, Firman K (2012). Mechanistic insight into Type I restriction endonucleases. Front Biosci. (Landmark Ed.).

[11] Cooper LP, Dryden DT (1994). The domains of a type I DNA methyltransferase. Interactions and role in recognition of DNA methylation. J. Mol. Biol..

[12] Morgan RD (2016). Novel m4C modification in type I restriction-modification systems. Nucleic Acids Res.

[13] Kennaway CK (2012). Structure and operation of the DNA-translocating type I DNA restriction enzymes. Genes Dev..

[14] Kennaway CK (2009). The structure of M.EcoKI Type I DNA methyltransferase with a DNA mimic antirestriction protein. Nucleic Acids Res.

[15] Meselson M, Yuan R (1968). DNA restriction enzyme from E. coli. Nature.

[16] Linn S, Arber W (1968). Host specificity of DNA produced by Escherichia coli, X. In vitro restriction of phage fd replicative form. Proc. Natl Acad. Sci. USA.

[17] Uyen NT (2009). The fragment structure of a putative HsdR subunit of a type I restriction enzyme from Vibrio vulnificus YJ016: implications for DNA restriction and translocation activity. Nucleic Acids Res.

[18] Obarska A (2006). Structural model for the multisubunit Type IC restriction-modification DNA methyltransferase M.EcoR124I in complex with DNA. Nucleic Acids Res.

[19] Lapkouski M (2009). Structure of the motor subunit of type I restriction-modification complex EcoR124I. Nat. Struct. Mol. Biol..

[20] Kim JS (2005). Crystal structure of DNA sequence specificity subunit of a type I restriction-modification enzyme and its functional implications. Proc. Natl Acad. Sci. USA.

[21] Grinkevich P (2018). Crystal structure of a novel domain of the motor subunit of the Type I restriction enzyme EcoR124 involved in complex assembly and DNA binding. J. Biol. Chem..

[22] Gao P, Tang Q, An X, Yan X, Liang D (2011). Structure of HsdS subunit from thermoanaerobacter tengcongensis sheds lights on mechanism of dynamic opening and closing of type I methyltransferase. PLoS One.

[23] Calisto BM (2005). Crystal structure of a putative type I restriction-modification S subunit from Mycoplasma genitalium. J. Mol. Biol..

[24] Liu YP (2017). Structural basis underlying complex assembly and conformational transition of the type I R-M system. Proc. Natl Acad. Sci. USA.

[25] Gao Y (2020). Structural insights into assembly, operation and inhibition of a type I restriction-modification system. Nat. Microbiol.

[26] McMahon SA (2009). Extensive DNA mimicry by the ArdA anti-restriction protein and its role in the spread of antibiotic resistance. Nucleic Acids Res.

[27] Walkinshaw MD (2002). Structure of Ocr from bacteriophage T7, a protein that mimics B-form DNA. Mol. Cell.

[28] Otwinowski Z, Minor W (1997). Processing of X-ray diffraction data collected in oscillation mode. Methods Enzymol..

[29] Minor W, Cymborowski M, Otwinowski Z, Chruszcz M (2006). HKL-3000: the integration of data reduction and structure solution-from diffraction images to an initial model in minutes. Acta Crystallogr D. Biol. Crystallogr.

[30] Adams PD (2010). PHENIX: a comprehensive Python-based system for macromolecular structure solution. Acta Crystallogr D. Biol. Crystallogr.

[31] Emsley P, Lohkamp B, Scott WG, Cowtan K (2010). Features and development of Coot. Acta Crystallogr D. Biol. Crystallogr.

[32] Krissinel E, Henrick K (2007). Inference of macromolecular assemblies from crystalline state. J. Mol. Biol..

[33] Winn MD (2011). Overview of the CCP4 suite and current developments. Acta Crystallogr D. Biol. Crystallogr.

[34] Hsiao K, Zegzouti H, Goueli SA (2016). Methyltransferase-Glo: a universal, bioluminescent and homogenous assay for monitoring all classes of methyltransferases. Epigenomics.

[35] Zhou J, Horton JR, Blumenthal RM, Zhang X, Cheng X (2021). Clostridioides difficile specific DNA adenine methyltransferase CamA squeezes and flips adenine out of DNA helix. Nat. Commun..

[36] Madeira F (2019). The EMBL-EBI search and sequence analysis tools APIs in 2019. Nucleic Acids Res.

[37] Kumar S, Stecher G, Li M, Knyaz C, Tamura K (2018). MEGA X: molecular evolutionary genetics analysis across computing platforms. Mol. Biol. Evol..

[38] Robert X, Gouet P (2014). Deciphering key features in protein structures with the new ENDscript server. Nucleic Acids Res.

