# Supplementary material for: Molecular insights into DNA recognition and methylation by non-canonical type I restriction-modification systems
Source: Nat Commun. 2022 Oct 27;13:6391. doi: 10.1038/s41467-022-34085-z (PMC9613975; doi:10.1038/s41467-022-34085-z)
Supplement: Supplementary file 1 — Supplementary Information [file 41467_2022_34085_MOESM1_ESM.pdf]

Supplementary Information

Molecular insights into DNA recognition and methylation by non-canonical type I restriction-modification systems

Jingpeng Zhu, Yina Gao, Yong Wang, Qi Zhan, Han Feng, Xiu Luo,

Songqing Liu, Hai Hou, Pu Gao

Supplementary Figures 1-11 and Legends

Supplementary Tables 1-3

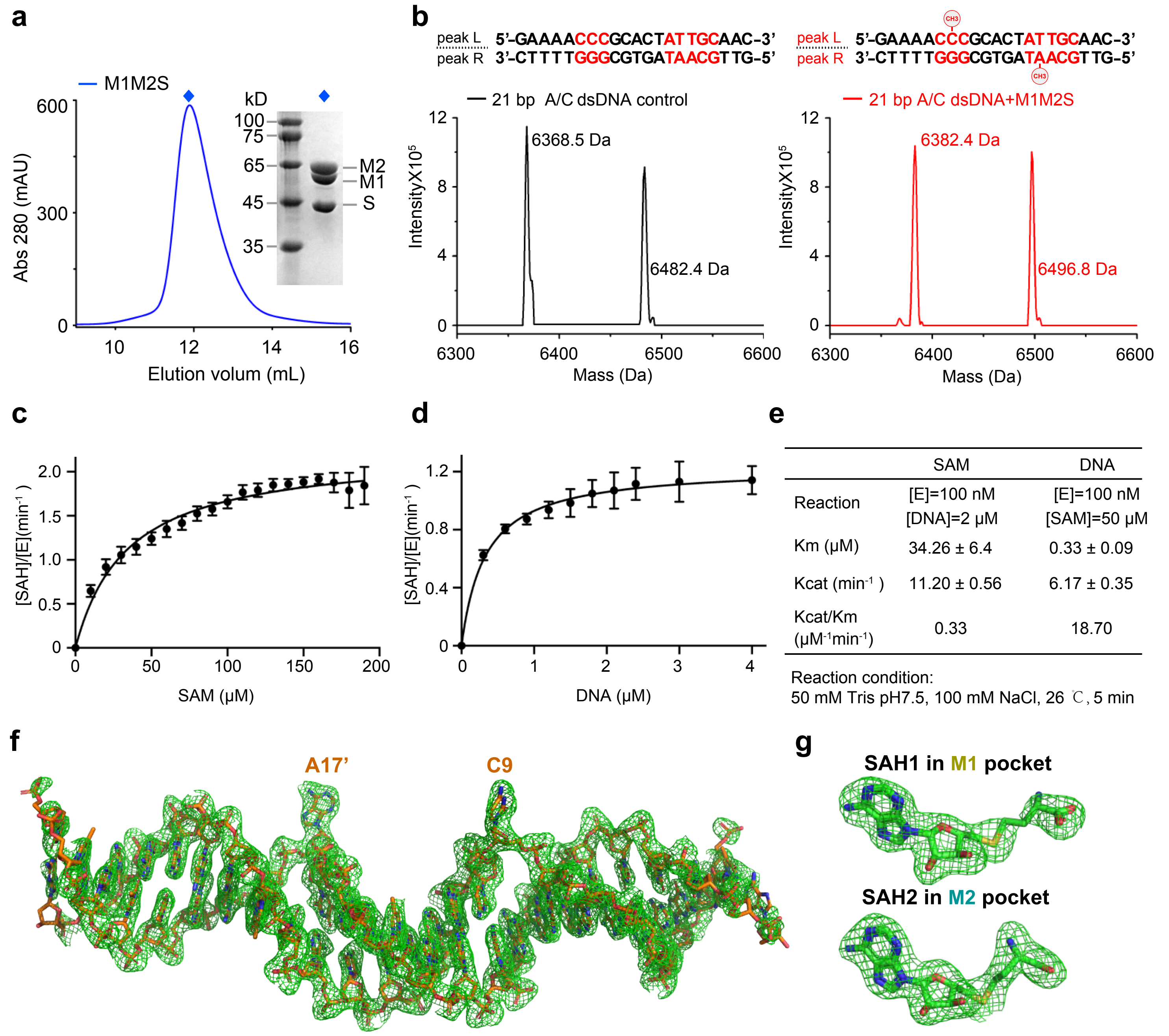

	SAM	DNA
Reaction	[E]=100 nM [DNA]=2 μM	[E]=100 nM [SAM]=50 μM
Km (μM)	34.26 ± 6.4	0.33 ± 0.09
Kcat (min^{-1})	11.20 ± 0.56	6.17 ± 0.35
Kcat/Km ($\mu M^{-1}min^{-1}$)	0.33	18.70

Reaction condition:
50 mM Tris pH7.5, 100 mM NaCl, 26 ℃, 5 min

Supplymentary Fig. 1 | Enzymatic activity of PacII MTase and electron density of DNA and SAH molecules.

a, Purification of PacII_M1M2S complex. Size exclusion chromatography on Superdex 200 10/300 column and SDS-PAGE result of the peak fraction (n=3).

b, Mass spectrometry analysis of the substrate DNA with (red) or without (black) wild-type enzyme (n=3).

c, d, The production of SAH was measured via a luminescence assay, and curves of [SAH]/[E] dependent on substrate concentration of methyl donor SAM (**c**) or DNA (**d**) was fitted according to the Michaelis-Menten equation (n= 3). Source data are provided as a Source Data file.

e, Summary of PacII MTase kinetic parameters. The mean ± SD represents three independent determinations (n=3).

f, g, mFo-Fc omit map (3σ) of DNA (**f**) and SAH molecules (**g**), the target base-flipped adenine and cytosine were visible.

Experiments in **a**, **c**, **d** were repeated independently three times with similar results. Source data are provided as a Source Data file.

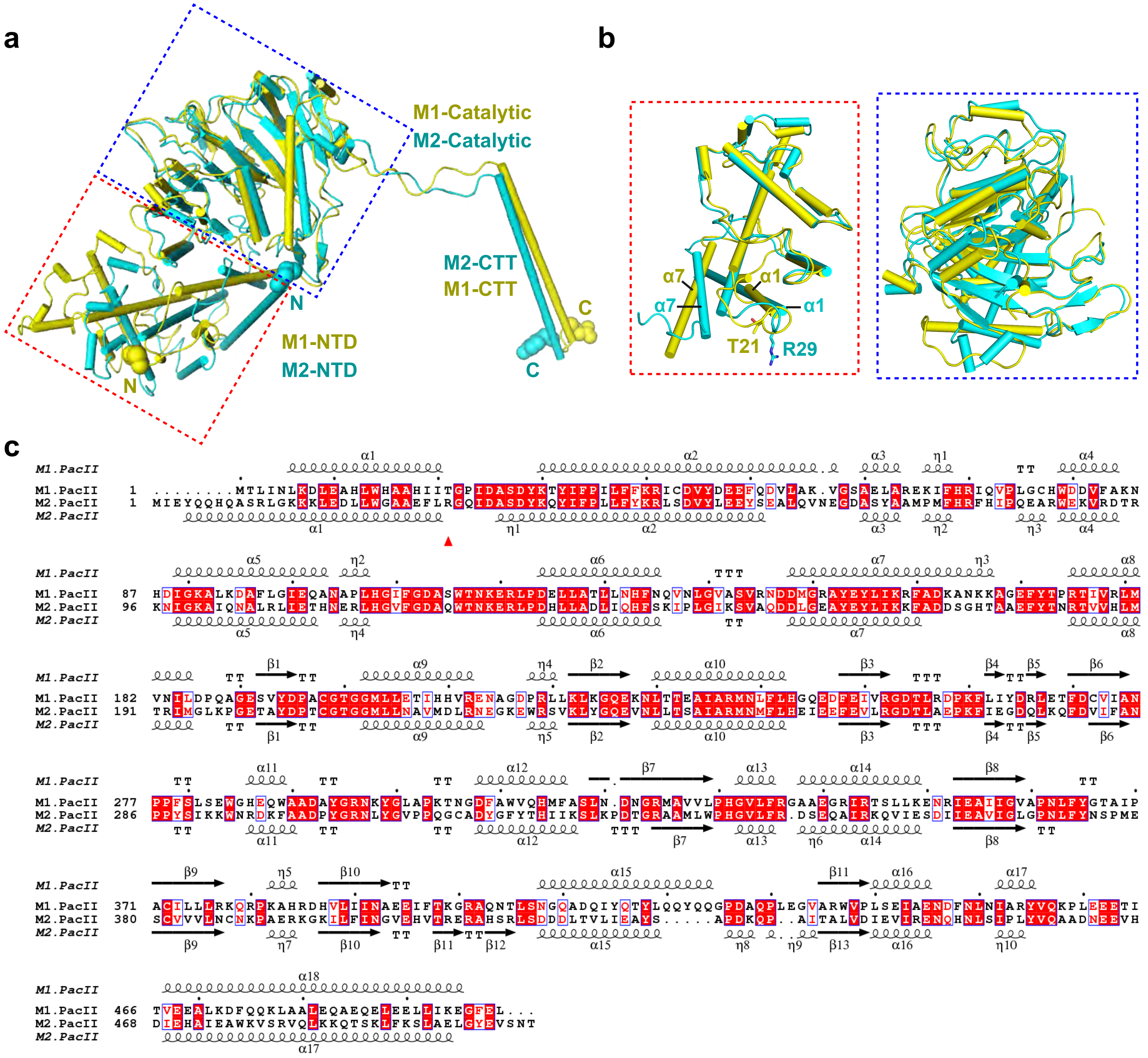

Supplymentary Fig. 2 | Structure comparison and sequence alignment of M1 and M2 subunits.

a, b, Overall structure (**a**) and individual domains (**b**) superimposition of M1 and M2 subunits.

c, Sequence alignment of full-length M1 and M2 subunits with secondary structure shown in up and down, respectively.

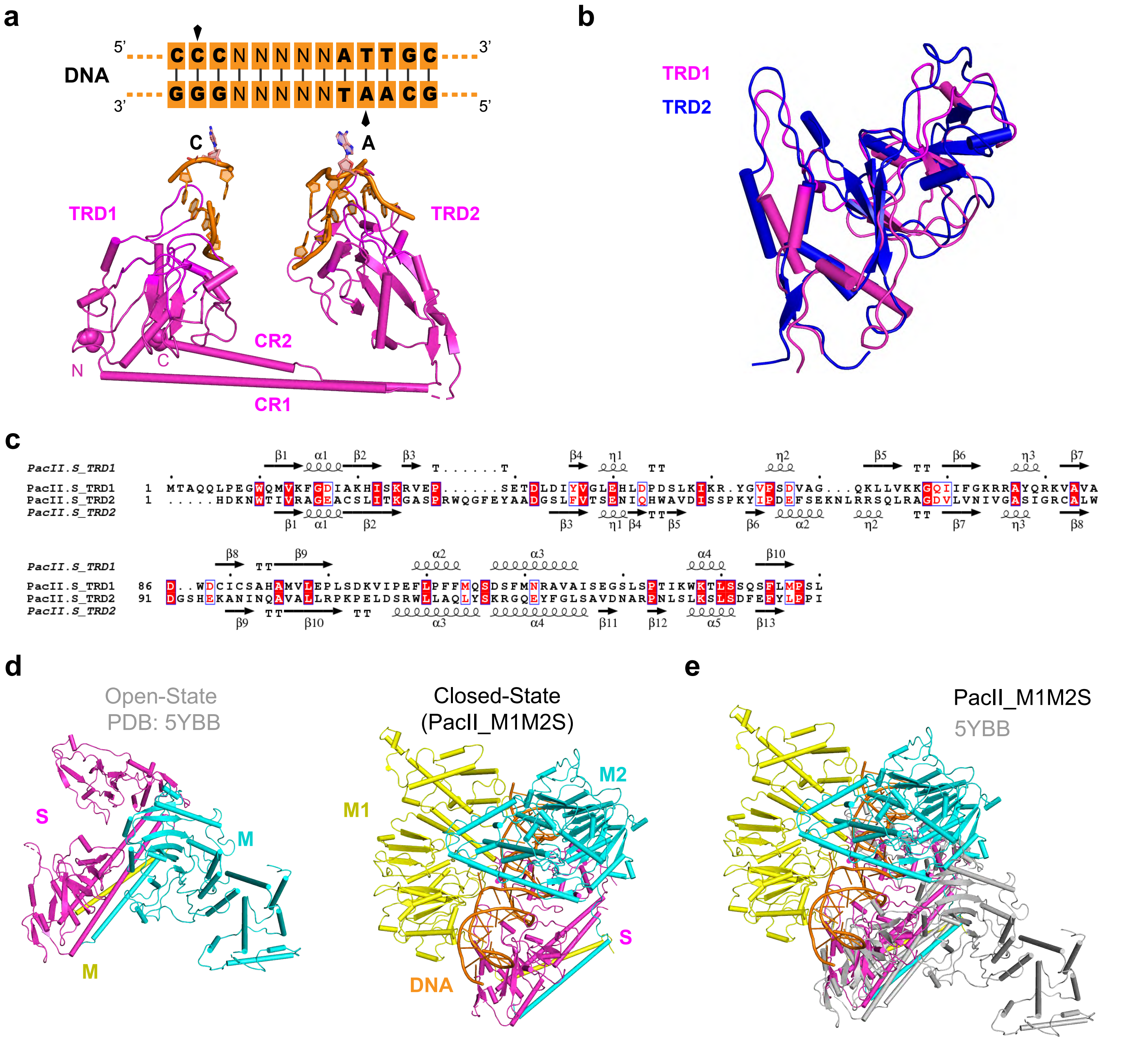

Supplymentary Fig. 3 | Comparison of two TRDs of S subunit, as well as open and closed type I MTases.

a, Structure of S subunit bound with bipartite specific DNA motifs.

b, Structural superimposition of TRD1 (magenta) and TRD2 (blue).

c, Sequence alignment of TRD1 and TRD2 with secondary structure shown in up and down, respectively.

d, Structures of the classical open-state type I M_2S (PDB: 5YBB) and the novel closed-state PacII_M1M2S-DNA-SAH. S subunits are shown in the same orientations in both structures.

e, Superimposition between the open-state and closed-state structures using the S subunits as references. PacII_M1M2S was color-coded as in (**d**) and the classical open-state type I M_2S (PDB: 5YBB) was shown in gray.

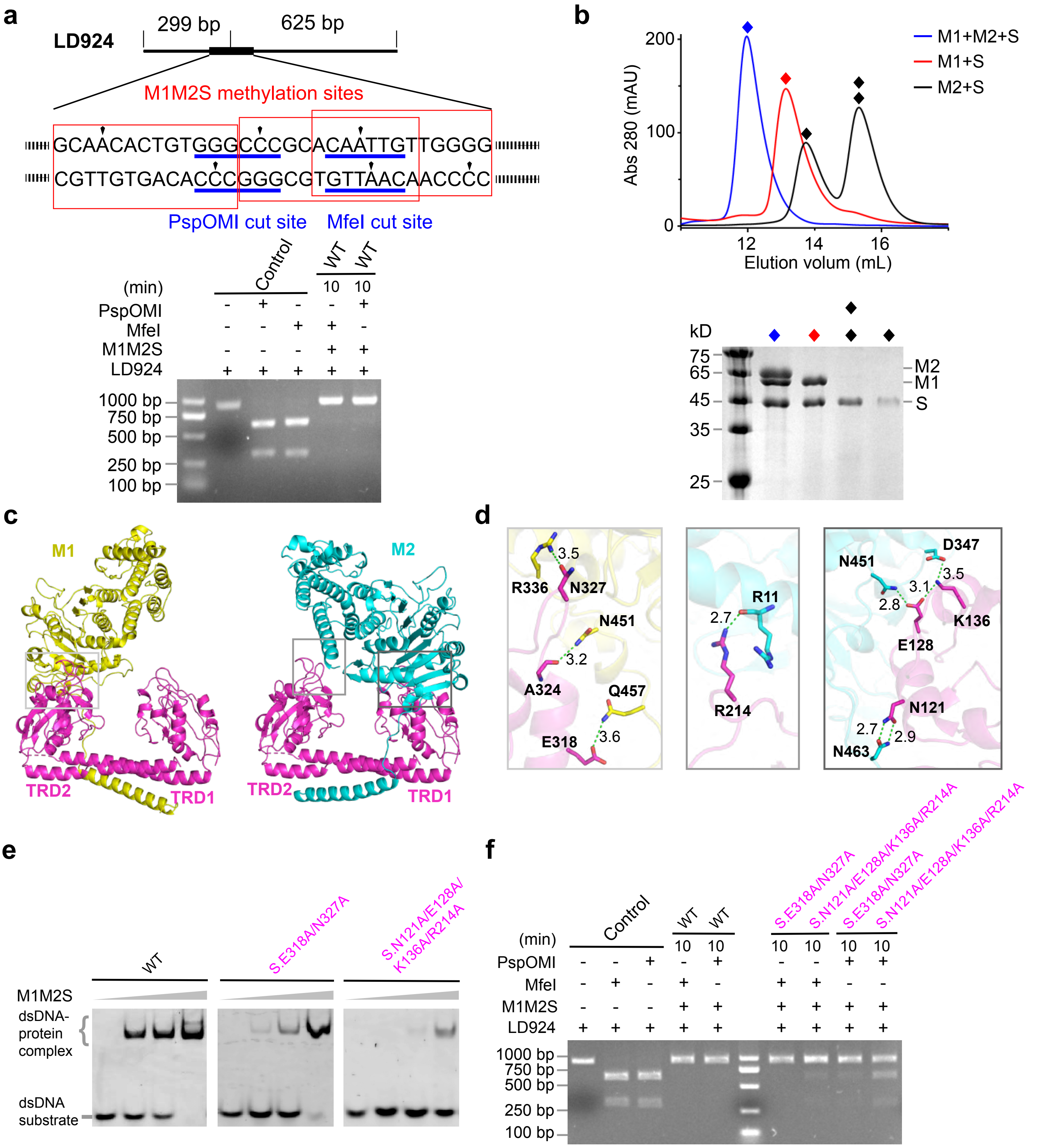

Supplymentary Fig. 4 | Assembly and asymmetric interactions of PacII_M1M2S complex.

a, A linear DNA (termed as LD924) containing three PacII recognition sits (up) and the cleavage assay by PspOMI/MfeI-digestion (down) (n=3).

b, Size exclusion chromatography (up) and SDS-PAGE results (down) of co-expressed samples including M1M2S, M1S, M2S. The S subunit alone exhibited a dynamic equilibrium of monomer and dimer (n=3).

c, d, Overall view (**c**) and detailed interactions (**d**) of M1-TRD2 and M2-TRD2/TRD1 interfaces. Green dashed lines indicate hydrogen bonds.

e, f, Electrophoretic mobility shift analysis (**e**) and PspOMI/MfeI-digestion analysis (**f**) of mutations within M1-TRD2 and M2-TRD2/TRD1 interfaces (n=3).

Experiments in **a**, **b**, **e**, **f** were repeated independently three times with similar results. Source data are provided as a Source Data file.

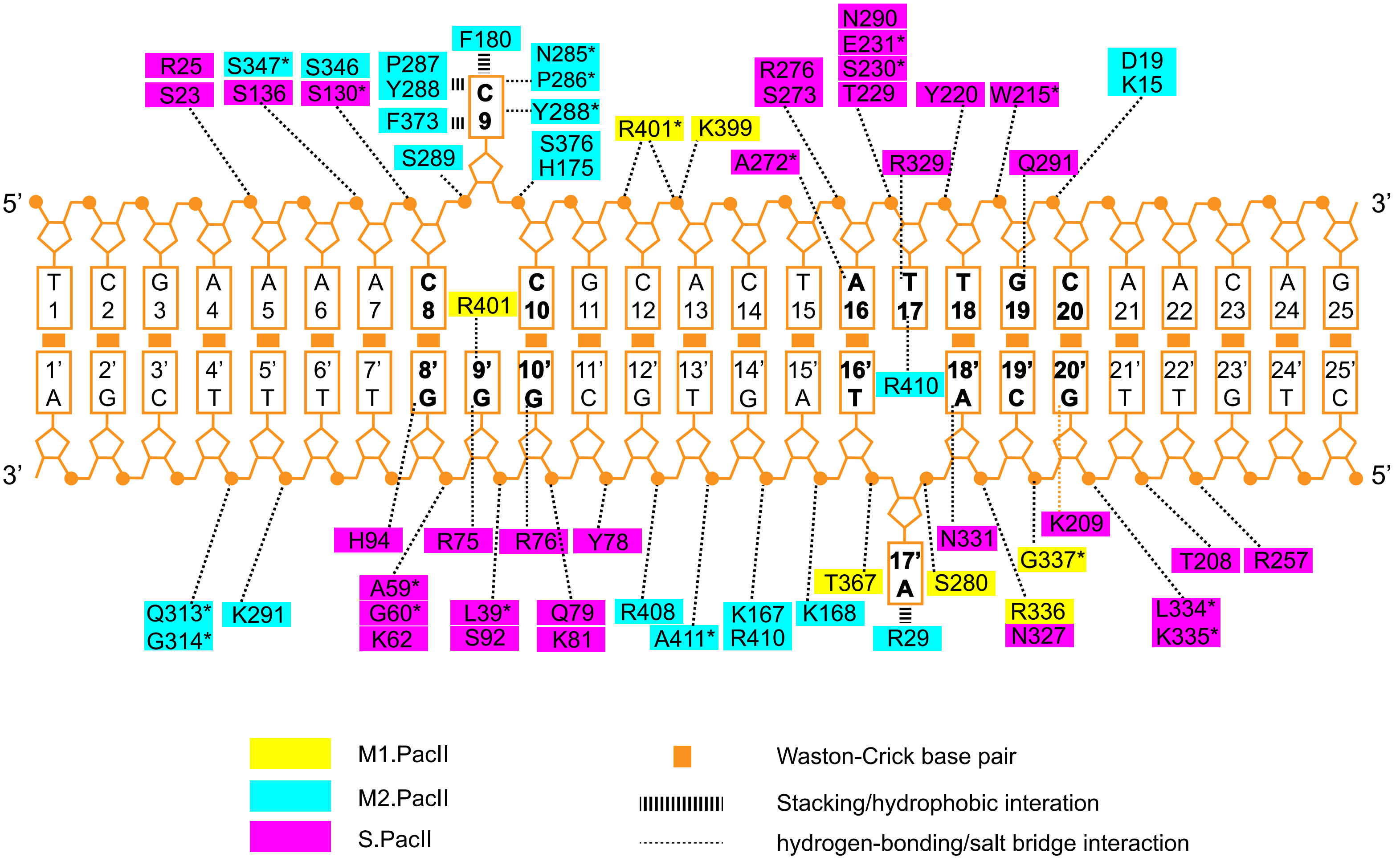

Supplymentary Fig. 5 | Schematic of intermolecular interactions between DNA and PacII_M1M2S heterotrimer complex. Residues from M1, M2, and S subunits are colored in yellow, cyan, and magenta, respectively (* means the mainchain of indicated residue). The bipartite specific DNA sequences recognized by S subunit are colored in bold black. Hydrogen bonds and salt bridges are shown in slight dotted lines. Stacking and hydrophobic interactions are shown in bold dotted lines. Watson-Crick base pairs are shown as orange squares.

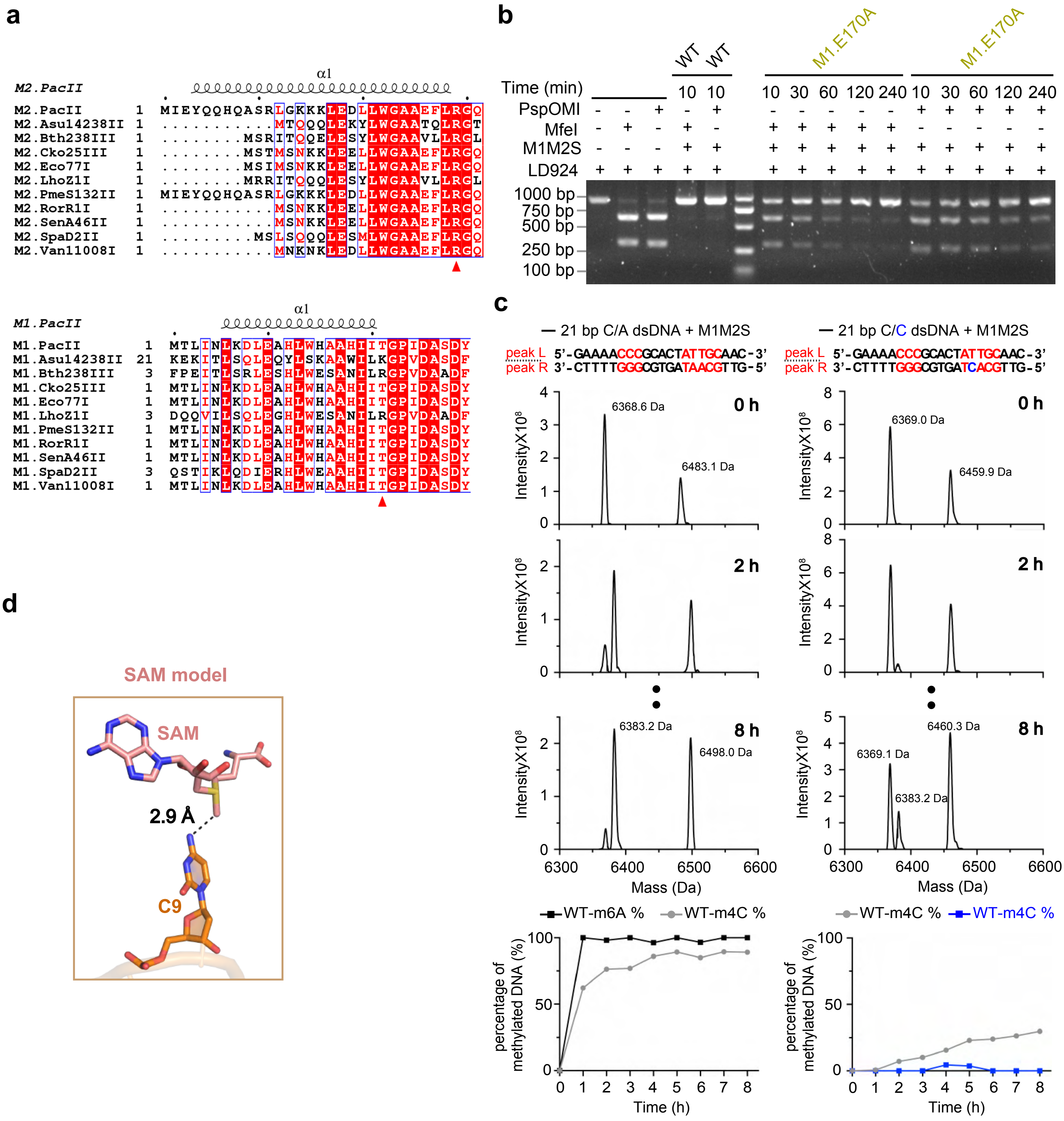

Supplymentary Fig. 6 | Asymmetric methylation analysis of M1 and M2 subunits.

a, Sequence alignment of helix α1 from candidate M1 and M2 subunits, respectively. The positions of M1_T21 and M2_R29 were highlighted by red triangles.

b, PspOMI/MfeI-digestion analysis of mutant M1_E170A (n=3).

c, Mass spectrometry analysis of original and mutant DNA (A17'C) substrates at different time points (0 to 8 h), proportions of m6A and m4C methylated DNA were counted and plotted, respectively.

d, The N4 atom of target cytosine (C9) within M2's catalytic pocket contacted with the modeled SAM molecule (2.9 Å, black dashed line).

Experiment in **b** was repeated independently three times with similar results. Source data are provided as a Source Data file.

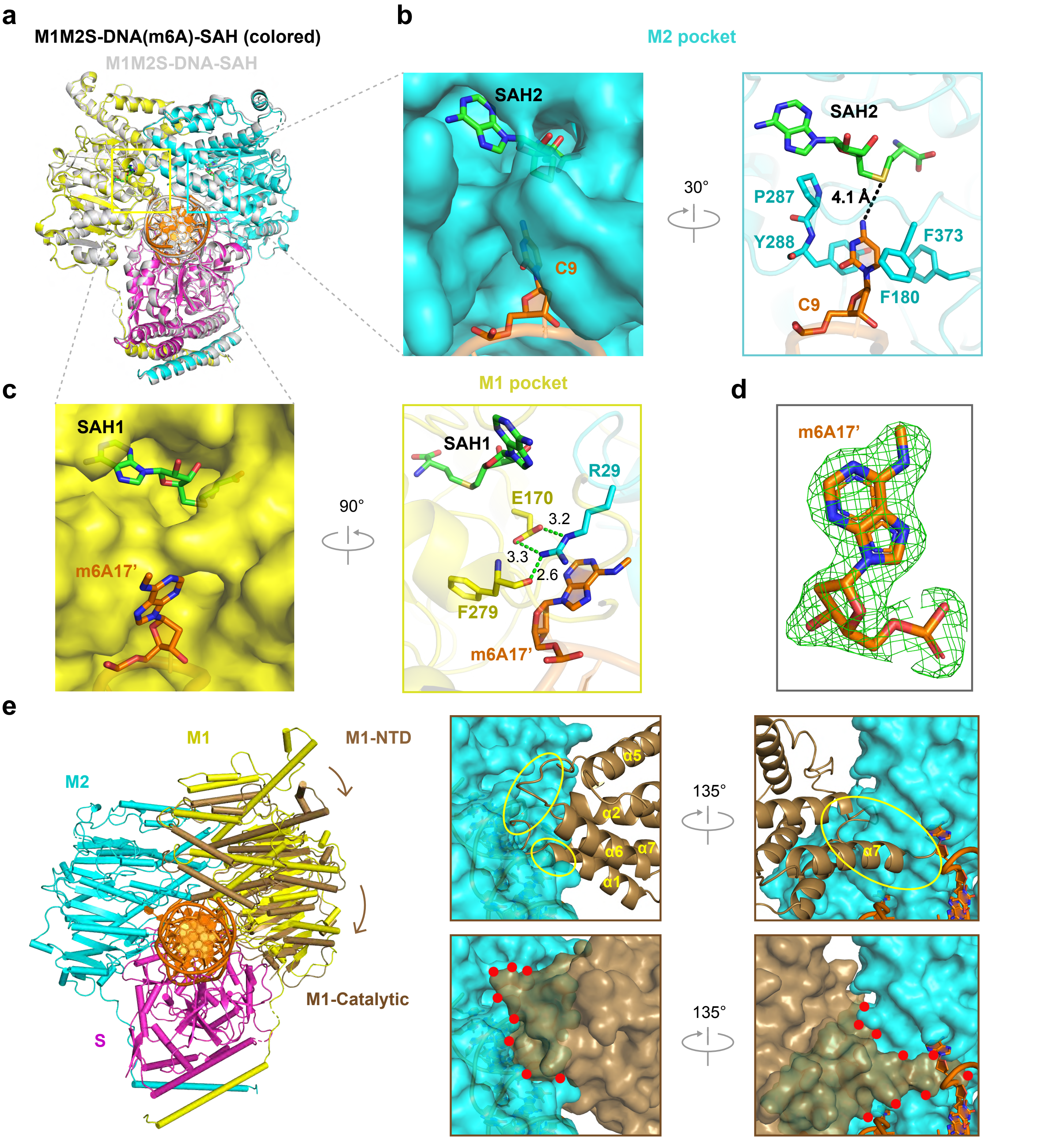

Supplymentary Fig. 7 | Catalytic pockets of M1M2S-DNA(m6A)-SAH complex, and lying-down structural model of M1 subunit.

a, Structural superimposition of M1M2S-DNA-SAH and M1M2S-DNA(m6A)-SAH complexes.

b, c, Surface views and detailed environments of the catalytic pockets within M2 (**b**) and M1 (**c**) subunits of M1M2S-DNA (m6A)-SAH complex.

d, mFo-Fc omit map (3σ) of target methylated adenine.

e, A modeled lying-down structure of M1 based on the current structure of M2 subunit, with the severe hindrance effects high-lighted as yellow circles and red dots.

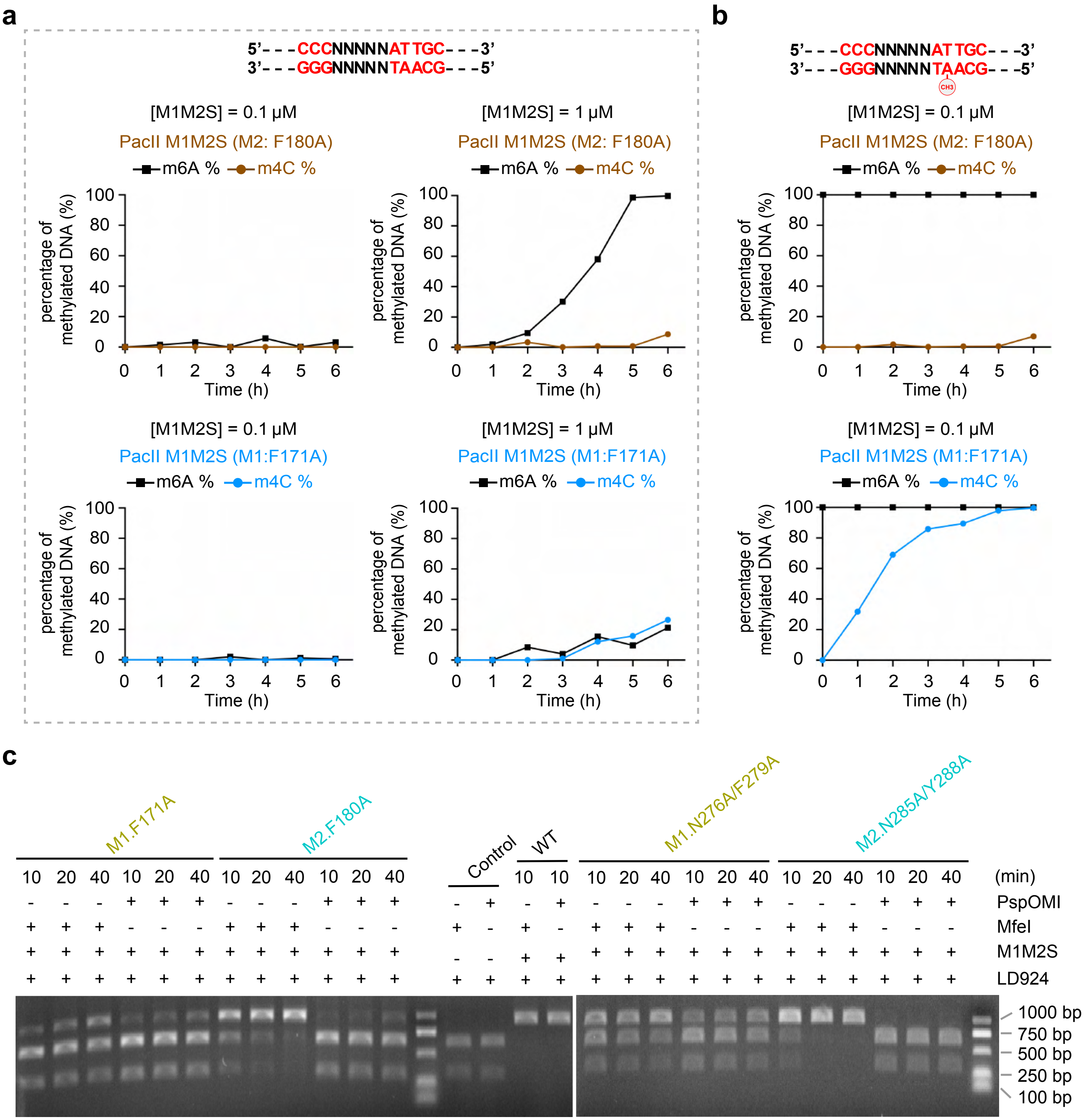

Supplymentary Fig. 8 | Mass spectrometry and PspOMI/MfeI-digestion analyses of catalytic pocket mutations within M1 and M2 subunits.

a, Two sets mass spectrometry analyses (low or high enzyme concentration) of target DNA at different time points (0-6 h), proportions of m6A and m4C methylated DNA were counted and plotted, respectively.

b, Mass spectrometry analysis (low enzyme concentration) of m6A methylated DNA at different time points (0-6 h), proportions of m6A and m4C methylated DNA were counted and plotted, respectively.

c, PspOMI/MfeI-digestion analysis of mutations within the catalytic of M1 (F171A, N276A/F279A) and M2 (F180A, N285A/Y288A) subunits (n=3).

Experiment in **c** was repeated independently three times with similar results. Source data are provided as a Source Data file.

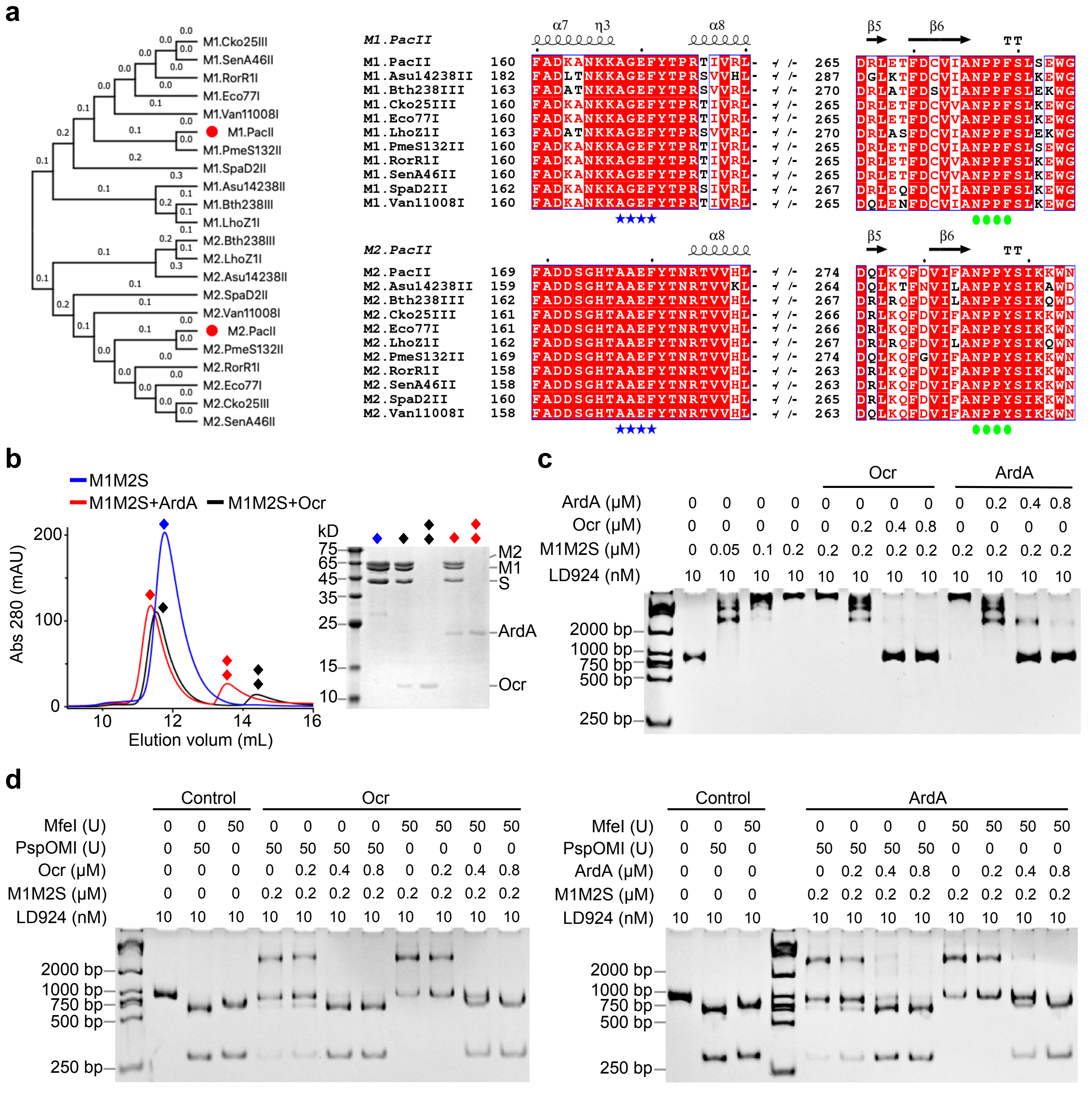

Supplymentary Fig. 9 | Conservative catalytic pockets of M1 and M2 subunits, and Ocr/ArdA-mediated inhibition on PacII_M1M2S complex.

a, Phylogenetic tree (numbers represent standardized distance scores) and sequence alignment of m6A/m4C Type I methyl-transferase of different species. The conserved regions were highlight in blue stars (M1: aa 168-171; M2: aa 177-180) and green dots (M1: aa 276-279; M2: aa 285-288).

b, Elution profiles of size exclusion chromatography and SDS-PAGE results for indicated samples (n=3).

c, Electrophoretic mobility shift analysis show that both Ocr and ArdA competed for the binding of PacII_M1M2S to DNA (n=3).

d, PspOMI/MfeI-digestion analysis show that both Ocr and ArdA inhibited the methylation activity of PacII_M1M2S (n=3).

Experiments in **b**, **c**, **d** were repeated independently three times with similar results. Source data are provided as a Source Data file.

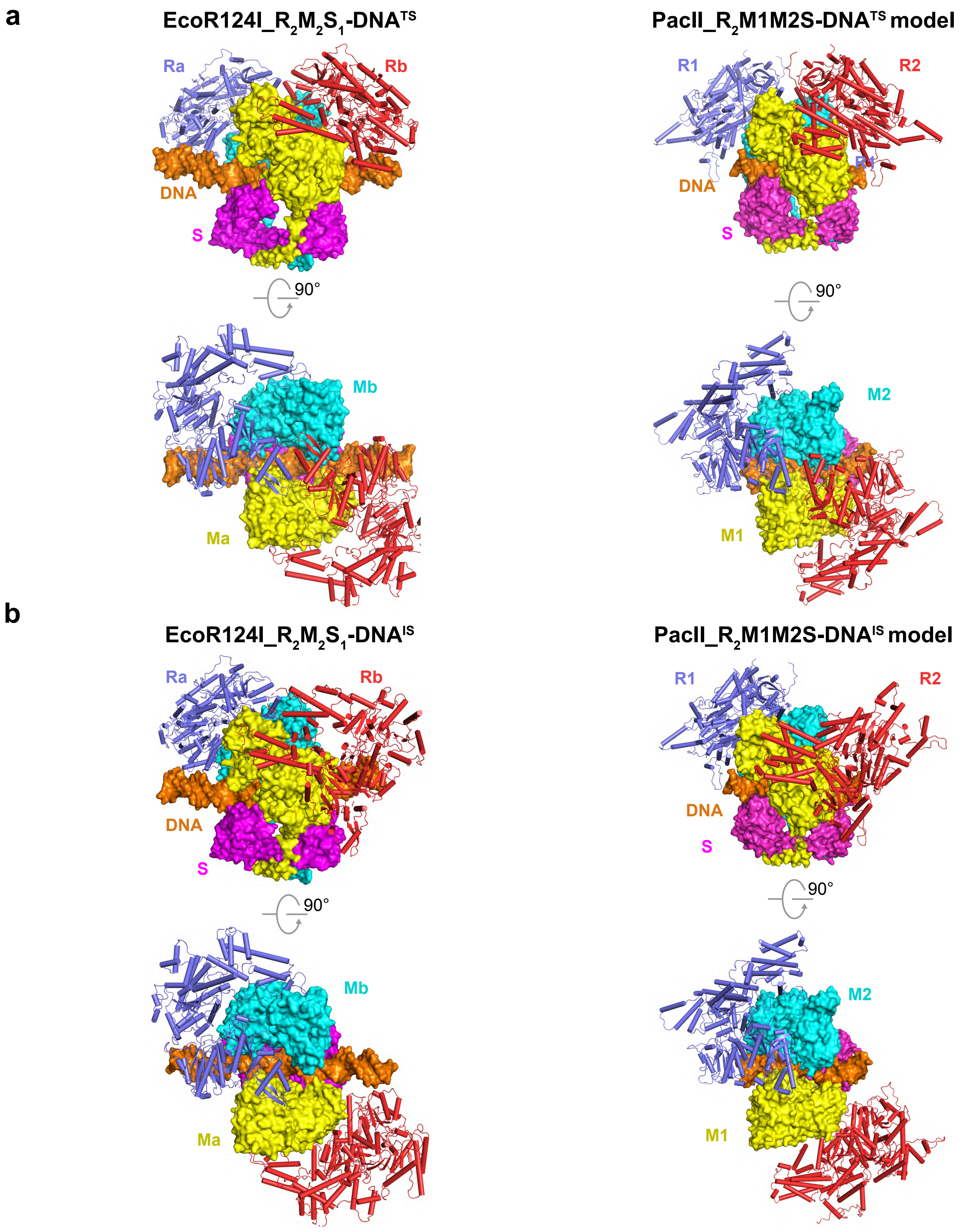

Supplymentary Fig. 10 | Potential models of PacII holoenzyme in two conformational states based on EcoR 124I.
a, Two views of the translocation state (TS) for EcoR124I_$R_2M_2S_1$ (left) and the predicted PacII_ R_2M1M2S holoenzyme (right).
b, Two views of the intermediate state (IS) for EcoR124I_ $R_2M_2S_1$ (left) and the predicted PacII_ R_2M1M2S holoenzyme (right).

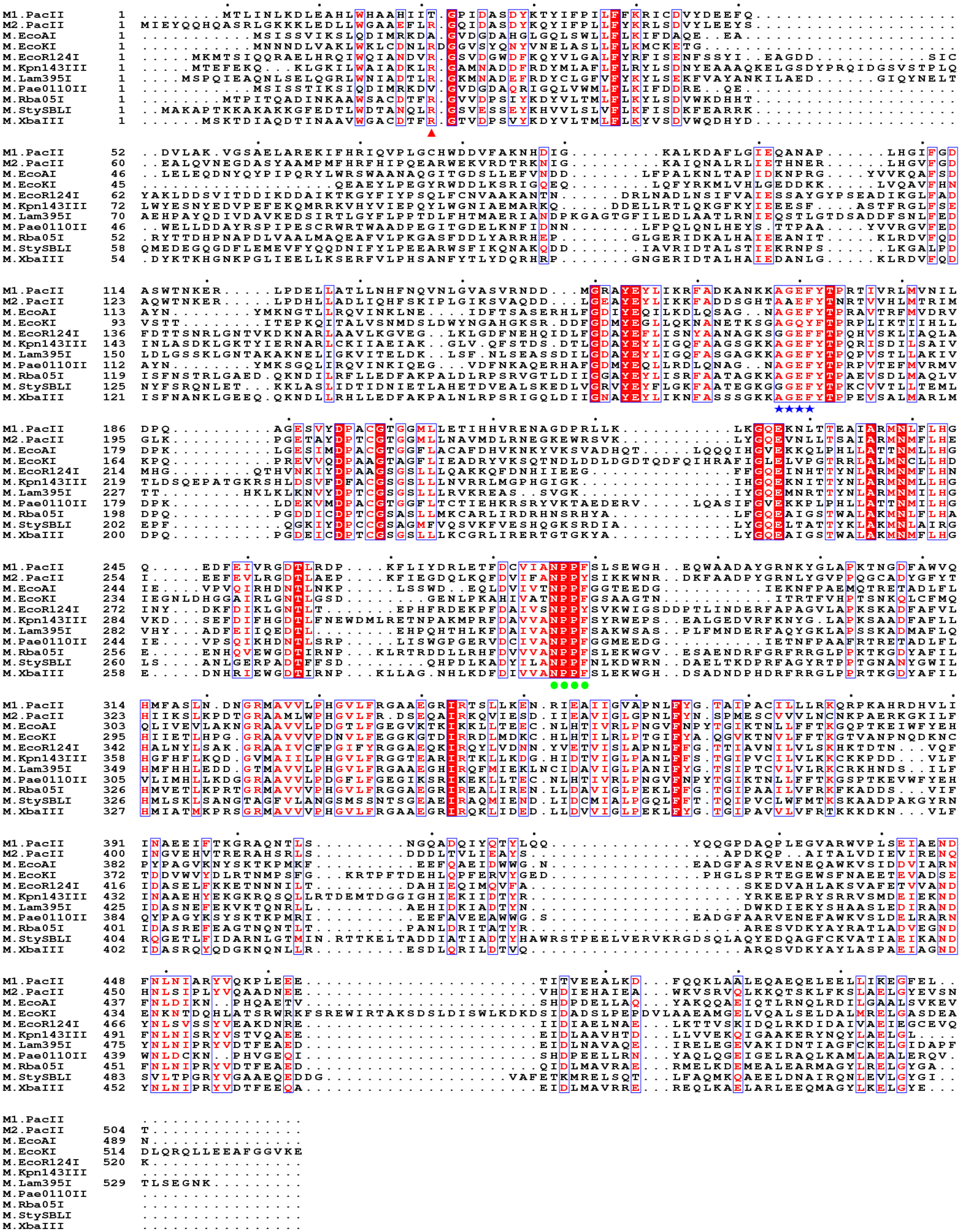

Supplymentary Fig. 11 | Sequence alignment of PacII_M1, PacII_M2, and different M subunits from classical m6A/m6A Type I R-M systems.

The conserved arginine residue, AGEF/AAEF motif and NPPF/NPPY motif were highlighted in red triangle, blue stars, and green dots, respectively.

Supplementary Table 1. Data collection and refinement statistics.

Crystal	M1M2S-DNA-SAH (7VRU)	M1M2S-DNA(m6A)-SAH (7VS4)
Beam line	SSRF-18U	SSRF-18U
Wavelength (Å)	0.979	0.979
Resolution range (Å)	41.24-2.40 (2.48-2.40)	49.17-2.55 (2.64-2.55)
Space group	$P\ 2_1\ 2_1\ 2_1$	$P\ 2_1\ 2_1\ 2_1$
Unit cell		
a, b, c (Å)	109.8 122.7 131.4	109.1 121.7 129.8
α, β, γ (°)	90 90 90	90 90 90
Total reflections	138658 (13395)	88024 (5361)
Unique reflections	69399 (6698)	56313 (5126)
Completeness (%)	99.29 (97.19)	98.84 (91.10)
Mean I/sigma(I)	17.54 (2.22)	18.54 (2.71)
R-merge	0.086 (0.905)	0.137 (1.048)
R-pim	0.031 (0.247)	0.023 (0.319)
CC1/2	0.998 (0.856)	1 (0.911)
CC*	1 (0.96)	1 (0.977)
Reflections used in refinement	69307 (6698)	56298 (5126)
Reflections used for R-free	3511 (331)	2773 (249)
R-work	0.189 (0.253)	0.184 (0.224)
R-free	0.234 (0.309)	0.240 (0.290)
Number of non-hydrogen atoms	12230	12392
macromolecules	11942	11964
ligands	SAH	SAH
solvent	288	428
Protein residues	1362	1365
RMS (bonds)	0.009	0.008
RMS (angles)	1.04	1.03
Ramachandran favored (%)	96.66	96.82
Ramachandran allowed (%)	3.34	3.18
Ramachandran outliers (%)	0.00	0.00

Statistics for the highest-resolution shell are shown in parentheses.

Supplementary Table 2. Novel m4C/m6A Type I R-M systems used for sequence alignments.

No.	organism	enzyme	Specificity of S subunit	Base Methylation of M subunits
1	*Pseudomonas alcaligenes*	PacII_M1M2S	5'... CCCNNNNNRTTGY ... 3'	M1-m6A/M2-m4C
2	*Aequorivita sublithincola*	Asu14238II_M1M2S	5'... GCCNNNNNNTCC ... 3'	M1-m6A/M2-m4C
3	*Burkholderia thailandensis* FDAARGOS_238	Bth238III_M1M2S	5'... CCCNNNNNCTGG ... 3'	M1-m6A/M2-m4C
4	*Citrobacter koseri* AR_0025	Cko25III_M1M2S	5'... CCCNNNNNRTCG ... 3'	M1-m6A/M2-m4C
5	*Escherichia coli* AR_0077	Eco77I_M1M2S	5'... CCCNNNNNCTC ... 3'	M1-m6A/M2-m4C
6	*Laribacter hongkongensis* HLGZ1	LhoZ1I_M1M2S	5'... GCCNNNNNCTCC ... 3'	M1-m6A/M2-m4C
7	*Pseudomonas mendocina* S13.2	PmeS132II_M1M2S	5'... CCCNNNNNNTGCG ... 3'	M1-m6A/M2-m4C
8	*Raoultella terrigena* R1Gly	RorR1I_M1M2S	5'... CCCNNNNNNTGAA ... 3'	M1-m6A/M2-m4C
9	*Salmonella enterica subsp. enterica serovar* Agona 460004 2-1	SenA46II_M1M2S	5'... CCCNNNNNRTAG ... 3'	M1-m6A/M2-m4C
10	*Salinispira pacifica* L21-RPul-D2	SpaD2II _M1M2S	5'... GCCNNNNNNTCG ... 3'	M1-m6A/M2-m4C
11	*Vibrio anguillarum* CNEVA NB11008	Van11008I_M1M2S	5'... GCCNNNNNNTGCT ... 3'	M1-m6A/M2-m4C

Supplementary Table 3. Classical Type I R-M systems used for sequence alignments.

No.	organism	enzyme	Specificity of S subunit	Base methylation of M subunits
1	*Arenibacter algicola* SMS7	AalSMS7III_M_2S	5'... GAGNNNNNNNGTG ... 3'	M-m6A/M-m6A
2	*Escherichia coli* 15T-	EcoAI_M_2S	5'... GAGNNNNNNNGTCA ... 3'	M-m6A/M-m6A
3	*Escherichia coli* K-12 substr. MG1655	EcoKI_M_2S	5'... AACNNNNNNGTGC ... 3'	M-m6A/M-m6A
4	*Escherichia coli* (R124)	EcoR124I_M_2S	5'... GAANNNNNNRTCG ... 3'	M-m6A/M-m6A
5	*Klebsiella pneumoniae* AR_0143	Kpn143III_M_2S	5'... ACGNNNNNGTTG ... 3'	M-m6A/M-m6A
6	*Lelliottia amnigena* FDAARGOS_395	Lam395I_M_2S	5'... CAAGNNNNNGGT ... 3'	M-m6A/M-m6A
7	*Pseudomonas aeruginosa* AR_0110	Pae0110II_M_2S	5'... CACNNNNNNNRTGT ... 3'	M-m6A/M-m6A
8	*Rhodobaca barguzinensis* alga05	Rba05I_M_2S	5'... TGGANNNNNNNTTC ... 3'	M-m6A/M-m6A
9	*Salmonella enterica* serovar blegdam	StySBLI_M_2S	5'... GGYANNNNNNTCG ... 3'	M-m6A/M-m6A
10	*Xanthomonas badrii*	XbaIII_M_2S	5'... CCAGNNNNNGGT ... 3'	M-m6A/M-m6A
